# A structure-function analysis shows SARS-CoV-2 BA.2.86 balances antibody escape and ACE2 affinity

**DOI:** 10.1016/j.xcrm.2024.101553

**Published:** 2024-05-08

**Authors:** Chang Liu, Daming Zhou, Aiste Dijokaite-Guraliuc, Piyada Supasa, Helen M.E. Duyvesteyn, Helen M. Ginn, Muneeswaran Selvaraj, Alexander J. Mentzer, Raksha Das, Thushan I. de Silva, Thomas G. Ritter, Megan Plowright, Thomas A.H. Newman, Lizzie Stafford, Barbara Kronsteiner, Nigel Temperton, Yuan Lui, Martin Fellermeyer, Philip Goulder, Paul Klenerman, Susanna J. Dunachie, Michael I. Barton, Mikhail A. Kutuzov, Omer Dushek, OPTIC Consortium, Elizabeth E. Fry, Juthathip Mongkolsapaya, Jingshan Ren, David I. Stuart, Gavin R. Screaton

**Affiliations:** 1 Chinese Academy of Medical Science (CAMS) Oxford Institute (COI), University of Oxford, Oxford, UK; 2 Centre for Human Genetics, Nuffield Department of Medicine, University of Oxford, Oxford, UK; 3 Division of Structural Biology, Nuffield Department of Medicine, University of Oxford, Centre for Human Genetics, Oxford, UK; 4 Centre for Free Electron Laser Science, Hamburg, Germany; 5 NIHR Oxford Biomedical Research Centre, Oxford University Hospitals NHS Foundation Trust, Oxford, UK; 6 Division of Clinical Medicine, School of Medicine and Population Health, University of Sheffield, Sheffield, UK; 7 Sheffield Teaching Hospitals NHS Foundation Trust, Sheffield, UK; 8 NDM Centre for Global Health Research, Nuffield Department of Medicine, University of Oxford, Oxford, UK; 9 Peter Medawar Building for Pathogen Research, University of Oxford, Oxford, UK; 10 Viral Pseudotype Unit, Medway School of Pharmacy, University of Kent and University of Greenwich Chatham Maritime, Kent ME4 4TB, UK; 11 Radcliffe Department of Medicine, John Radcliffe Hospital, University of Oxford, Oxford, UK; 12 Department of Paediatrics, University of Oxford, Oxford, UK; 13 Translational Gastroenterology Unit, Nuffield Department of Medicine, University of Oxford, Oxford, UK; 14 Mahidol-Oxford Tropical Medicine Research Unit, Mahidol University, Bangkok, Thailand; 15 Sir William Dunn School of Pathology, Oxford, UK; 16 Diamond Light Source Ltd, Harwell Science & Innovation Campus, Didcot, UK

**Keywords:** BA.2.65, coronavirus, SARS-CoV-2, antigenic escape, virus structure, ACE2 binding, virus evolution, receptor binding

## Abstract

BA.2.86, a recently described sublineage of SARS-CoV-2 Omicron, contains many mutations in the spike gene. It appears to have originated from BA.2 and is distinct from the XBB variants responsible for many infections in 2023. The global spread and plethora of mutations in BA.2.86 has caused concern that it may possess greater immune-evasive potential, leading to a new wave of infection. Here, we examine the ability of BA.2.86 to evade the antibody response to infection using a panel of vaccinated or naturally infected sera and find that it shows marginally less immune evasion than XBB.1.5. We locate BA.2.86 in the antigenic landscape of recent variants and look at its ability to escape panels of potent monoclonal antibodies generated against contemporary SARS-CoV-2 infections. We demonstrate, and provide a structural explanation for, increased affinity of BA.2.86 to ACE2, which may increase transmissibility.

## Highlights

• BA.2.86, highly mutated from BA.2, is mapped in the landscape of recent variants
• It is marginally less evasive than XBB.1.5 but escapes a panel of BA.2 antibodies
• It has increased affinity for ACE2, possibly aiding transmission
• Its RBD is primed for further escape at residues 455 and/or 456, as seen in JN.1

## Graphical abstract

**Figure:**
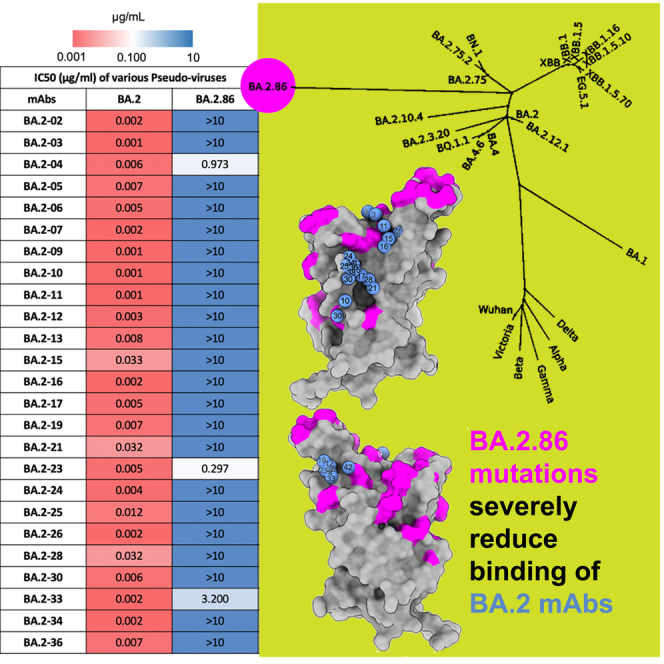

## Teaser

BA.2.86 spike has evolved extensively from the early Omicron variant BA.2, allowing it to escape the vast majority of anti-BA.2 monoclonal antibodies. While it is not quite as evasive as some other recent variants such as XBB.1.5, it has high affinity for the virus receptor ACE2 and is primed for further antigenic escape by mutation at residues 455 and/or 456 of the spike.

## Introduction

The majority of the human population is believed to have been exposed to SARS-CoV-2 by natural infection (773 million cases and 7 million deaths confirmed as of 02/01/24 https://covid19.who.int/, but the actual numbers are likely much higher) and/or vaccination, often on multiple occasions. This herd immunity has put the SARS-CoV-2 genome under huge selective pressure to evade pre-existing immune responses, hence the abundance of variants (https://www.cdc.gov/coronavirus/2019-ncov/variants/variant-classifications.html).

A particular hotspot for mutational change in SARS-CoV-2 is in the spike gene, encoding the spike protein (S).^1^ The characteristic spikes on the surface of coronaviruses are formed by trimers of S, linked to the virion through transmembrane helices at the C terminus. S is made up of an N-terminal S1 domain, responsible for attachment to the host receptor angiotensin converting enzyme 2 (ACE2),^2^ and a C-terminal S2 domain, which through conformational rearrangement executes fusion of host and viral membranes, allowing entry of viral RNA into the host cell cytoplasm, initiating the infectious cycle.^3^

S1 contains a string of rather small domains, including the N-terminal domain (NTD) and receptor binding domain (RBD). The RBD is positioned at the top of S and can adopt a range of conformational states, from a fully exposed “up” conformation, able to interact with ACE2, to a more hidden “down” conformation. At the tip of the RBD is a small 25 amino acid (aa) patch, the receptor binding motif, that forms a landing pad for ACE2.^2^

Characterization of panels of monoclonal antibodies (mAbs) from previously infected donors has allowed a detailed mapping of the antigenic determinants for potent virus neutralization and enabled the generation of a number of mAbs for therapeutic or prophylactic use.^4^^,^^5^^,^^6^^,^^7^^,^^8^^,^^9^^,^^10^^,^^11^ Antibodies binding to the so-called super-site in the NTD^12^ do not antagonize interaction with ACE2, but can show potent neutralization; these antibodies and their function are poorly understood. The RBD is the binding site for a number of potent mAbs,^7^^,^^9^^,^^10^ many of which bind on, or in close proximity to, the ACE2 binding surface and block ACE2 interaction.^2^ Another group, characterized by mAb S309 bind distant to the ACE2 binding surface, in proximity to the N-linked glycan attached to N343; these do not block ACE2 interaction and may function to destabilize the S trimer.^4^

The NTD and RBD are hotspots for mutational change, either by substitution or, in the case of the NTD, the insertion or deletion of amino acid residues.^1^^,^^13^^,^^14^ For the NTD, it is likely that mutation is in part immune driven, with the majority of potent anti-NTD mAbs being specific to a single or limited number of lineages.^15^ For the RBD, mutations can increase the affinity for ACE2, potentially giving the virus a transmission advantage.^16^ Mutations at the binding sites for neutralizing anti-RBD antibodies can lead to a reduction of the neutralizing titers of immune serum, promoting immune escape and enabling reinfection.^17^ Mutations of key residues in the ACE2 interaction surface can therefore act as a double-edged sword for the virus, potentially modulating ACE2 affinity at the same time as causing antibody escape.

**Figure 1:**
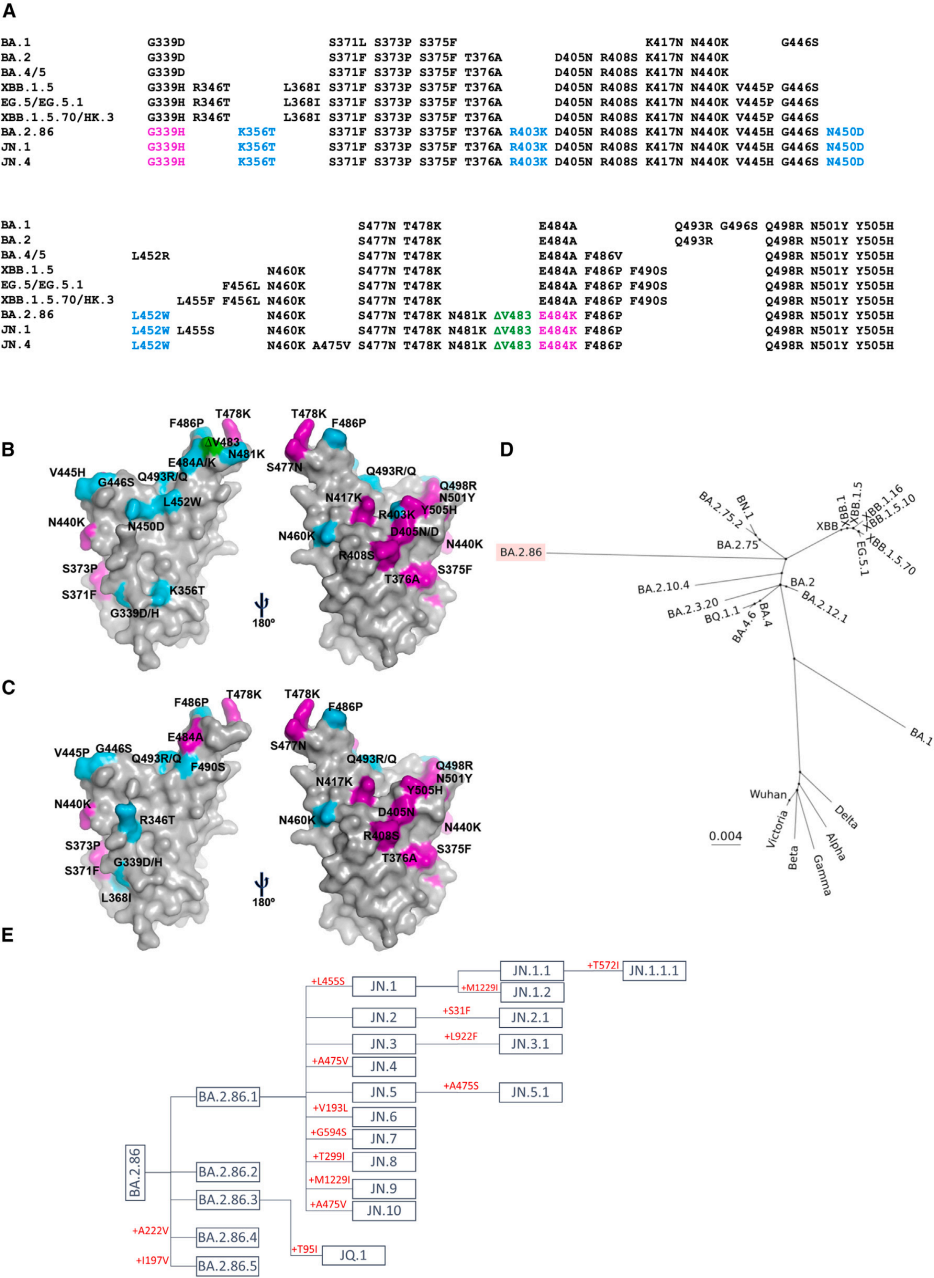
Sequence changes in BA.2.86 compared with other Omicron sublineages (A) Sequence alignments of BA.2.86 RBD with Omicron sublineages BA.1, BA.2, BA.4/5, XBB.1.5, EG.5/EG.5.1, XBB.1.5.70/HK.3, JN.1, and JN.4. (B) Surface representation of BA.2.86 mutations shown on BA.2 RBD. (C) XBB.1.5 mutations on BA.2 RBD. Mutations in common are colored in magenta, further mutations in BA.2.86 and XBB.1.5 in cyan, and V483 deletion in BA.2.86 in green. If there are two letters after the residue number in the labels, the first letter indicates the residue type for BA.2 and the second BA.2.86 or XBB.1.5. (D) Phylogenetic tree generated by aligning spike sequences of the SARS-CoV-2 variants. (E) Evolutionary tree of BA.2.86 with spike mutations indicated in red. See also Figure 2 and Table S1.

The first sequence for BA.2.86 was deposited on August 13, 2023 (EPI_ISL_18096761) from Israel and, since then, 32,029 sequences have been deposited from multiple countries that belong to BA.2.86 lineage. BA.2.86 contains 51 aa substitutions, 8 aa deletions, and 4 aa insertions compared with the ancestral Wuhan S sequence.^18^ It does not appear that BA.2.86 has arisen from the currently dominating strains related to XBB and the closest ancestor is BA.2^19^ (Figure 1). The large jump from BA.2 (38 aa changes in S alone) and the lack of any intermediate sequences, has led to speculation that BA.2.86 may have emerged in an immunosuppressed individual chronically infected with BA.2.^20^^,^^21^ The emergence, global spread, and the ability of BA.2.86 to cause outbreaks such as that reported in a care home in the UK with a 86.6% attack rate among residents (https://www.cidrap.umn.edu/covid-19/uk-reports-nursing-home-covid-outbreak-involving-ba286-variant) has led to concern that it may show increased immune escape and be poised to cause a new wave of infection. WHO added BA.2.86 and JN.1 (BA.2.86 + L455S) to SARS-CoV-2 variants of interest list on November 21 and December 13, 2023, respectively. According to a GISAID report from December 26, JN.1 is the second dominant strain in North America after HV.1, but dominant in the remaining four regions: Europe, Asia, Oceania, and South America.

Here, we characterize BA.2.86 using a panel of sera collected following natural infection or vaccination and demonstrate that it shows less antibody evasion than several other contemporary strains allowing us to place BA.2.86 on an antigenic map. We also look at the ability of a panel of potent (against XBB.1.5) human mAbs isolated following infection with contemporary SARS-CoV-2 strains to neutralize BA.2.86, showing that the majority of these potent mAbs can still neutralize BA.2.86 and provide structural explanations for this cross-reactivity. However, these potent antibodies have focused their footprints to a distinct epitope on the RBD where they are vulnerable to escape by mutation at residues 455 and 456. Indeed, two BA.2.86 sublineages have already emerged that challenge these antibodies: JN.1 (BA.2.86 + L455S) and JN.4 (BA.2.86 + A475V). Finally, we measure the affinity of BA.2.86 RBD for ACE2 and show a 2.2-fold increase in affinity compared with XBB.1.5, for which we provide a structural explanation. In summary, while the mutations acquired by BA.2.86 do not impart a step change in antibody escape, the increase in ACE2 affinity may give BA.2.86 a transmission advantage. In practice, further modest changes, such as the acquisition of the L455S mutation to form the JN.1 variant, have already led to a sharp increase in infections making JN.1 the globally dominant strain, highlighting the power of a single mutation and indicating that BA.2.86 is likely the start point for further evolution.

## Results

### The BA.2.86 lineage

BA.2.86 has assembled a unique suite of mutations and appears to have evolved separately from the XBB sublineage of Omicron, which recently dominated infections worldwide (https://gisaid.org/hcov-19-variants-dashboard/). Compared with S from the ancestral Wuhan strain, there are 63 aa changes present in BA.2.86, with 51 substitutions, 8 deletions, and 4 insertions. There are hotspots of mutation in the NTD and RBD, known sites for the binding of potent antibodies. In the NTD there are 13 substitutions, 7 deletions, and 4 insertions (7.9% change compared with Wuhan), and in the RBD 24 substitutions and 1 deletion (12.9% change compared with Wuhan) (Figures 1A–1C). Of particular interest is the deletion of valine at position 483, which immediately precedes the ACE2 footprint residue E484.

A phylogenetic tree (Figure 1D) places BA.2.86 far distant from other Omicron lineages with its likely origin BA.2, which has not been a major circulating sublineage for more than a year, having been replaced by BA.4/5 in mid-2022 (https://cov-spectrum.org/explore/World/AllSamples/AllTimes/variants?nextcladePangoLineage=ba.4∗&). Compared with BA.2, there are 38 aa changes in BA.2.86, 29 substitutions, 5 deletions, and 4 insertions; with 9 substitutions, 4 deletions, and 4 insertions in NTD and 12 substitutions and 1 deletion in RBD. The evolutionary tree of BA.2.86 (Figure 1E) shows new sublineages allocated to the BA.2.86 family that have acquired mutations in S. JN.1 is BA.2.86 + L455S and is currently the predominant BA.2.86 variant in four out of five regions globally.

There has been very extensive evolution of the virus from BA.2. The absence of any intermediate species in the BA.2.86 sublineage leads to speculation that it may have evolved over a long period in a chronically BA.2-infected immunosuppressed individual, where the accrual of multiple mutations and their potential admixture by viral recombination events has led to a virus fit to escape into the general population and spread globally. It is therefore interesting to note that changes at all but one (the insertion of the peptide MPLF at residue 16 in the highly mutable NTD) of the mutated residues, despite their independent evolution, have been observed in other contemporary variants derived from BA.2, showing extreme evolutionary convergence (Table S1). Deletions in the ACE2 binding surface are rare but deletion of V483 was seen in some sequences in 2022 and there have been a total of 25,992 sequences with this deletion deposited since the start of the pandemic. Five of the mutations from BA.2 in the BA.2.86 RBD—V445H, L452W, V483del, E484K, and F486P—have shown more than one mutation in other variants.

### Neutralization of BA.2.86 lineage by vaccine serum

We constructed a panel of pseudotyped lentiviruses^22^^,^^23^ expressing the S gene of a series of variants from Omicron sublineages, BA.2, BA.4/5, XBB.1.5, EG.5 (XBB.1.5 + F456L), EG.5.1 (EG.5 + Q52H), XBB.1.5.70 (EG.5 + L455F), HK.3 (XBB.1.5.70 + Q52H), BA.2.86, JN.1 (BA.2.86 + L455S), and JN.4 (BA.2.86 + A475V) (https://gisaid.org/hcov19-variants/). EG.5.1 and HK.3 were included to represent more contemporary variants but, as seen in the results, Q52H did not have a significant impact on neutralization. Neutralization assays were performed using serum collected 18 months following a third dose of vaccine (Pfizer-BioNtech or Moderna, *n* = 17), and 6 months after a fourth dose of vaccine (Bivalent Pfizer-BioNtech [Wuhan/BA.1] or Bivalent Moderna [Wuhan/BA.1], *n* = 23) (Table S2A).

**Figure 2:**
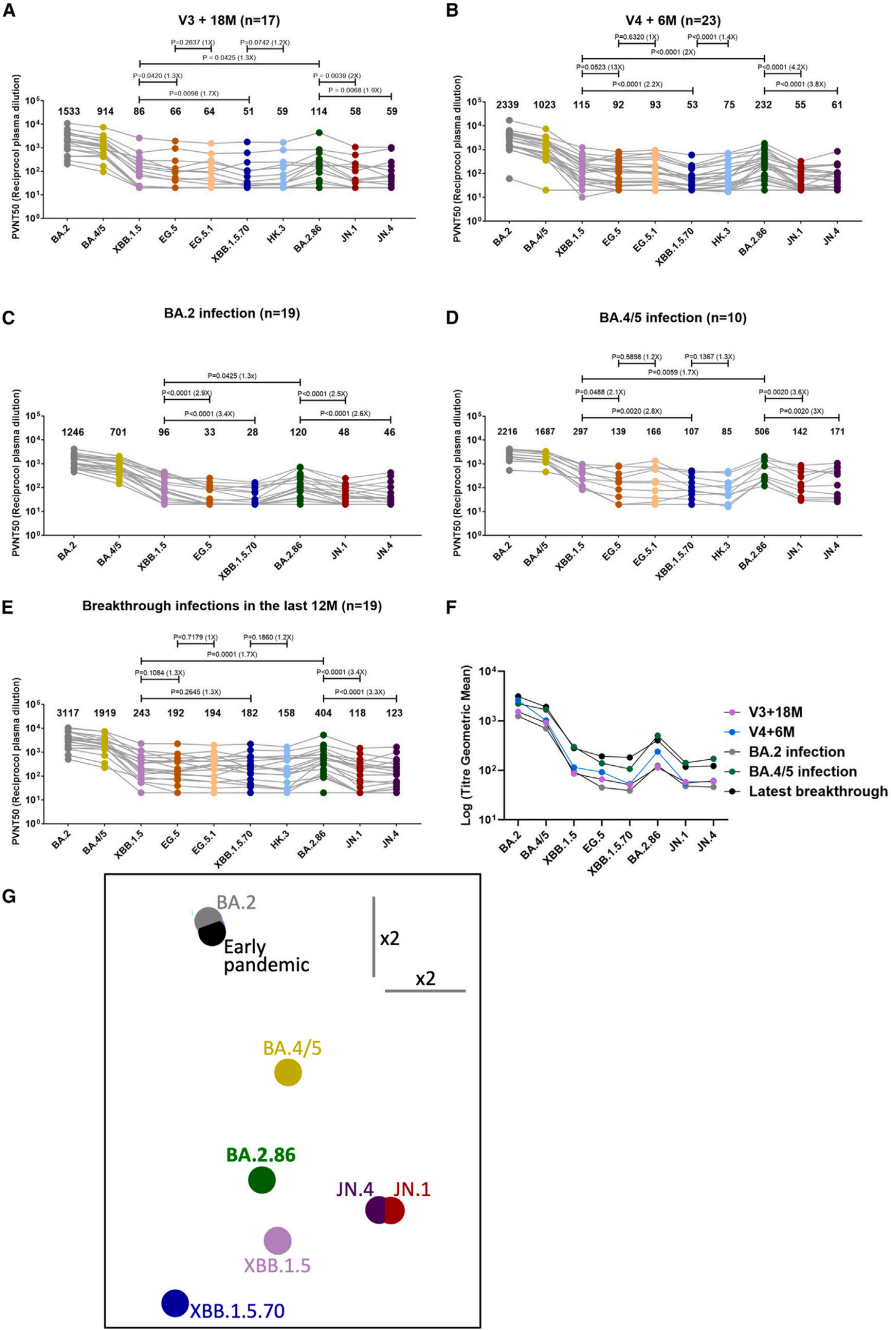
Pseudoviral neutralization assays of BA.2.86 by vaccine and infected serum samples (A and B) Geometric mean PVNT50 values for the indicated viruses using serum obtained from vaccinated volunteers after 18 months (*n* = 17), neutralization assays were performed in duplicate and the average titer was taken after a third dose of Pfizer BNT162b2 or Moderna vaccine (A) and 6 months after a fourth bivalent vaccine dose (*n* = 23) (B). (C–E) Serum from vaccinees suffering breakthrough infections by (C) BA.2 (*n* = 19), (D) BA.4/5 (*n* = 10), and (E) a set of samples collected following vaccine breakthrough infections in the last year (*n* = 19). (F) A composite figure for the geometric means of all serum samples against selected Omicron sublineages. The Wilcoxon matched-pairs signed rank test was used for the analysis and two-tailed *p* values were calculated. (G) Antigenic map showing BA.2.86 in the context of the positions of previous lineages including several Omicron-related sublineages calculated from pseudovirus neutralization data. The distance between two positions is proportional to the reduction in neutralization titer when one of the corresponding strains is challenged with serum derived by infection by the other (see STAR Methods). We have previously described the method^24^; however, while in previous reports we generated a 3D map here we were able to describe the map in 2D with minimal impact on the target function (starting error function for random positions was 1.25, final errors were 0.038 and 0.039 for 3D and 2D models, respectively). An approximate scale bar is shown, the scale of the map is linear and is the same in all directions. See also STAR Methods and Figure 1.

For samples obtained 18 months after triple vaccination, geometric mean neutralization titers are shown above each column and BA.2.86 shows a 1.3-fold (*p* = 0.0425) increase compared with XBB.1.5, but titers of JN.1 and JN.4 show 2-fold (*p* = 0.0039) and 1.9-fold (*p* = 0.0068) decrease compared with BA.2.86, respectively. EG.5 (XBB.1.5 + F456L) and XBB.1.5.70 (EG.5 + L455F) show 1.3-fold (*p* = 0.0420) and 1.7-fold (*p* = 0.0098) reduction compared with XBB.1.5, respectively (Figure 2A).

Neutralization titers of the samples collected 6 months after a fourth bivalent dose of vaccine showed a similar trend, BA.2.86 titers show 2.0-fold (*p* < 0.0001) increase compared with XBB.1.5, but titers of JN.1 and JN.4 show 4.2-fold (*p* < 0.0001) and 3.8-fold (*p* < 0.0001) decrease compared with BA.2.86, respectively. EG.5 (XBB.1.5 + F456L) and XBB.1.5.70 (EG.5 + L455F) show 1.3-fold (*p* = 0.0523) and 2.2-fold (*p* < 0.0001) reduction compared with XBB.1.5, respectively (Figure 2B).

### Neutralization of BA.2.86 by sera collected following natural infection

Breakthrough BA.2 serum samples were taken from vaccinated volunteers ≥12 days from symptom onset (median 29 days; *n* = 19) and tested against various pseudotyped lentiviruses (Figure 2C). The geometric mean neutralization titers are shown above each column and BA.2.86 shows a 1.3-fold (*p* = 0.0425) increase compared with XBB.1.5, but titers of JN.1 and JN.4 show 2.5-fold (*p* < 0.0001) and 2.6-fold (*p* < 0.0001) decrease compared with BA.2.86, respectively. EG.5 (XBB.1.5 + F456L) and XBB.1.5.70 (EG.5 + L455F) show 2.9-fold (*p* < 0.0001) and 3.4-fold (*p* < 0.0001) reduction compared with XBB.1.5, respectively.

BA.4/5 serum samples taken from 10 individuals (all but one vaccinated) more than 14 days (median = 38 days) (Figure 2D) post BA.4/5 infection, show that BA.2.86 has 1.7-fold (*p* = 0.0059) increase compared with XBB.1.5, but titers of JN.1 and JN.4 show 3.6-fold (*p* = 0.0020) and 3-fold (*p* = 0.0020) decrease compared with BA.2.86, respectively. EG.5 (XBB.1.5 + F456L) and XBB.1.5.70 (EG.5 + L455F) show 2.1-fold (*p* = 0.0488) and 2.8-fold (*p* = 0.0020) reduction compared with XBB.1.5, respectively.

A set of recent breakthrough infection samples obtained from 19 vaccinated volunteers who had documented infections with several variants (Table S2B) between August 2022 and February 2023 follow the same trend (Figure 2E). BA.2.86 neutralization titers show that BA.2.86 has 1.7-fold (*p* = 0.0001) increase compared with XBB.1.5, but titers of JN.1 and JN.4 show 3.4-fold (*p* < 0.0001) and 3.3-fold (*p* < 0.0001) decrease compared with BA.2.86, respectively. EG.5 (XBB.1.5 + F456L) and XBB.1.5.70 (EG.5 + L455F) show 1.3-fold (*p* = 0.1084) and 1.3-fold (*p* = 0.2645) reduction compared with XBB.1.5, respectively.

In terms of neutralization titer BA.2, BA.4/5 and latest breakthrough infection serum, EG.5.1, and HK.3 show similar titers to EG.5 and XBB.1.5.70 (Figures 2C–2E).

In summary, neutralization of BA.2.86 by vaccinated or naturally infected serum is reduced compared with BA.2 and BA.4, but modestly increased compared with XBB.1.5, EG.5 (EG.5.1), and XBB.1.5.70 (HK.3); however, acquisition of mutations L455F and A475V in JN.1 and JN.4 leads to a marked reduction in neutralization titers compared with BA.2.86. The concordant results in all groups may result from immune imprinting in the participants, all but one of whom had been vaccinated in the early phase of the pandemic (Figure 2F).

### Antigenic cartography of BA.2.86

Neutralization data presented in Figures 2A–2F were merged with a library of neutralization data generated from vaccinated cases and from previous infection with ancestral virus, Alpha, Beta, Gamma, Delta, and BA.1,^7^^,^^17^^,^^25^^,^^26^^,^^27^ and an antigenic map was produced using our previously reported methodology.^28^ The distance between different isolates represents the antigenic distance between them, which is a measure of the reduction in neutralization titer when serum raised to one variant is used to neutralize a different variant (Figure 2G). Note that a subset of the variants is displayed for clarity.

When Omicron BA.1 first emerged, it was placed far distant on the antigenic map from the previous variants Wuhan, Alpha, Beta, Gamma, and Delta.^27^ The current map demonstrates the scale of evolution of SARS-CoV-2 since the emergence of BA.1 and BA.2. The evolution of XBB and its sublineages have pushed the antigenic distance further still, with EG.5 and XBB.1.5.70 being the most distant of the lineages studied to date. As expected from the neutralization data presented in Figures 2A–2E, BA.2.86 occupies an intermediate space among contemporary variants.

### Neutralization by a panel of potent mAbs generated from BA.2-infected cases

**Figure 3:**
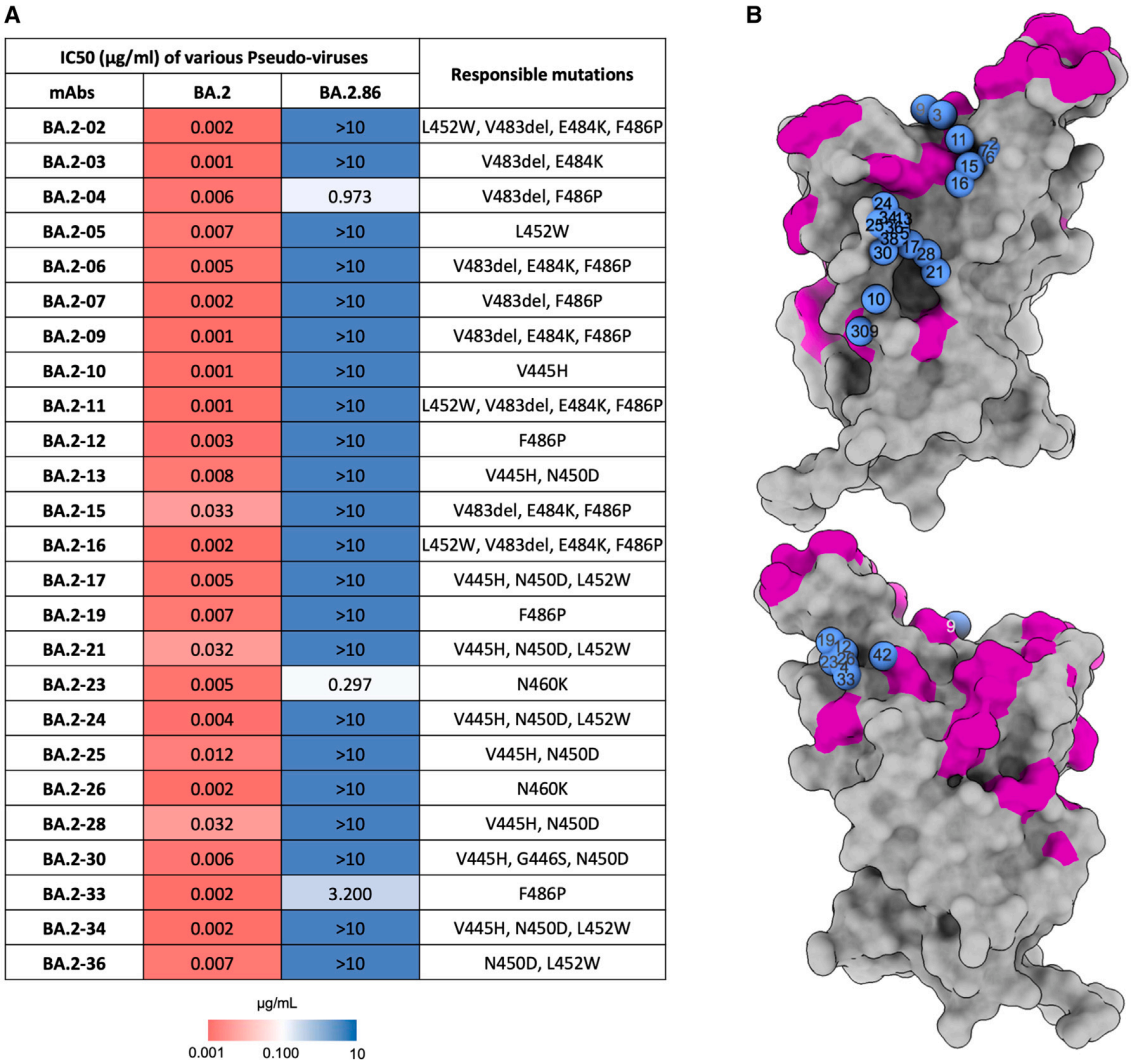
Pseudoviral neutralization assays using BA.2-specific monoclonal antibodies (A) Heatmap of BA.2.86 IC_50_ neutralization titers of 25 potent human mAbs made following BA.2 infection. BA.2 neutralization titers are taken from Dijokaite-Guraliuc et al.^29^ The likely BA.2.86 mutations leading to loss of activity in BA.2.86 for each mAb are indicated in the final column. (B) BA.2 mAb binding positions (blue spheres) mapped on RBD surface by BLI competition measurements and structure determinations.^29^ RBD shown in gray surface representation with BA.2.86 mutation sites colored in magenta. S309^4^ and Omi-42 are also shown for reference.^9^ See also Figure S1.

Following the BA.2 wave of infection in early 2022 we generated a panel of 25 potent human mAbs (IC_50_ < 100 ng/mL) from infected volunteers.^29^ All these mAbs potently cross-neutralized the early pandemic strain (Victoria) and, as all participants in this study had been vaccinated, we speculated that these potent BA.2 neutralizing mAbs may have been generated from memory B cell clones laid down in the initial response to vaccination. Strikingly, the neutralization of all 25 mAbs was dramatically reduced against BA.2.86, with complete knockout of neutralizing activity of 22/25 mAbs (IC_50_ > 10 μg/mL) (Figure 3A).

We have previously mapped the binding sites of these 25 mAbs^29^ either by direct crystallographic determination or by imputing their binding sites using a BLI competition mapping technique (Figure 3B), with sentinel mAbs with structurally determined coordinates, which gave a precision of ∼8 Å.^7^ This, together with structural information for some BA.2 mAbs, allows us to propose which amino acid changes led to the failure of each antibody (see Figure 3A).

### Neutralization of BA.2.86 by a panel of mAbs potently neutralizing XBB.1.5

We generated a panel of mAbs from vaccinated individuals who suffered BA.4, BA.5.1, or XBB.1.5 infections. Memory B cells from six breakthrough infection cases were stained with XBB.1.5 RBD. In total, 127 RBD-specific antibodies were recovered and, following RT-PCR, mAbs were expressed and tested in neutralization assays against XBB.1.5. Only 10 mAbs, with IC_50_ neutralization titers <100 ng/mL to XBB.1.5 were selected for further study, which we refer to as XBB-1 to XBB-10 (Table S3).

**Figure 4:**
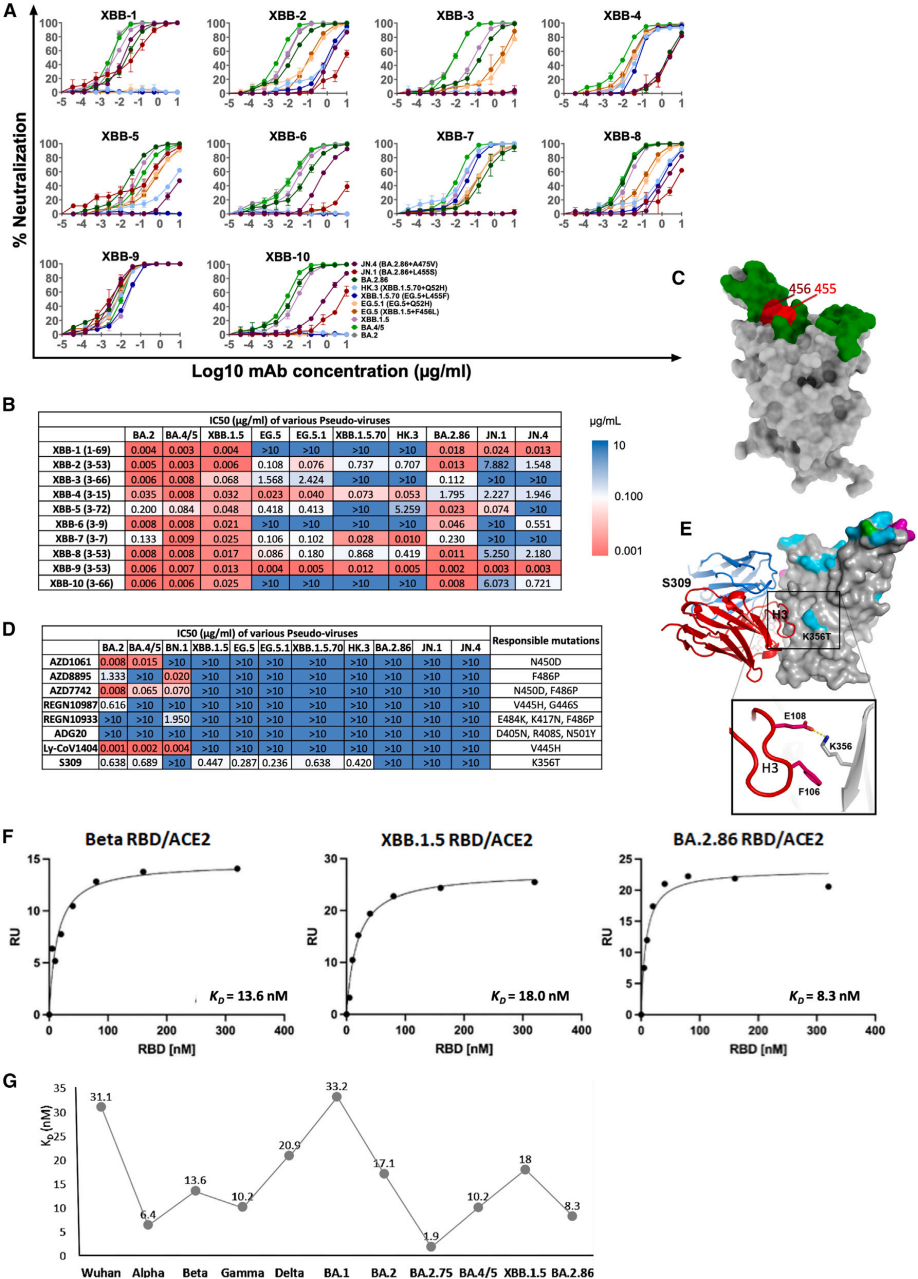
Neutralization curves for XBB.1.5 RBD-specific mAbs XBB-specific mAb isolated from breakthrough infection with recent variants. (A and B) (A) Titration curves for BA.2.86 are compared with BA.2, BA.4/5, XBB.1.5, EG.5, EG.5.1, XBB.1.5.70, HK.3, JN.1, and JN.4. Assays were performed twice in duplicate. Data are presented as mean values ± SEM. IC_50_ titers are shown as a heatmap with numbers in parentheses indicating heavy chain V genes in (B). (C) Surface representation of RBD with ACE2 footprint colored in green and the sites of mutations L455F and F456L highlighted in red (F456L in EG.5, L455F + F456L in XBB.1.5.70). (D) Heatmap of IC_50_ neutralization titers of mAbs developed for clinical use. (E) Binding pose of S309 (sotrovimab) and its interactions with K356.^4^ (F) Measurement of the affinity of ACE2 with BA.2.86, XBB.1.5, and Beta RBDs by surface plasmon resonance. Titration curves for ACE2 that flowed over the indicated immobilized RBDs are shown together with the calculated K_D_ values. (G) Comparison of ACE2/RBD affinities for RBDs from different SARS-CoV-2 variants. See also Figures 5, 6, and S2 and Tables S2 and S3.

All mAbs, except XBB-5 and XBB-7, showed potent cross-neutralization of BA.2 and BA.4/5 (Figures 4A and 4B). However, neutralization of EG.5 and XBB.1.5.70, containing F456L and L455F + F456L mutations in the RBD, respectively, were knocked out or reduced >10-fold compared with neutralization of XBB.1.5 in 7/10 of the potent mAbs. The focus of potent mAbs from recently infected individuals, on an epitope containing residues 455 and 456, on the back of the left shoulder of the RBD (Figure 4C), was likely because mAbs that bind to other epitopes on the RBD have had their neutralizing activities knocked out by the numerous mutations in successive SARS-CoV-2 variants, whereas the 455/456 region has remained more or less unscathed until recently (Figure 4C). It is notable that EG.5 (F456L) was the dominant Omicron sublineage in many regions such as the USA, China, and Japan, accounting for more than 25% of global cases until about November 2023, when JN.1 took over with an exponential increase in cases and now accounts for 32.6% of global cases (https://public.tableau.com/app/profile/raj.rajnarayanan/viz/ConvergentLineages-VariantSoup-World/G20).

Interestingly, BA.2.86 does not contain mutations at residues 455 or 456, likely explaining the higher neutralization titers against vaccine and naturally infected sera compared with EG.5 and XBB.1.5.70, and the neutralization titers of the XBB mAbs are comparable with those against XBB.1.5, with only XBB-4 showing >10-fold reduction in titer against BA.2.86 compared with XBB.1.5. However, mutations L455S in JN.1 and A475V in JN.4 substantially reduce the activity of most XBB mAbs compared with BA.2.86, leaving only mAb XBB-1 and XBB-9 retaining full activity.

Finally, we looked at the neutralization titers of mAbs developed for clinical use against BA.2.86, and the activity of all of them was completely knocked out, including S309/sotrovimab,^4^ which had maintained some activity against previously encountered variants apart from BN.1 (Figure 4D). It is likely that the K356T mutation in BA.2.86 abolishes the neutralization ability of S309, since this residue makes a salt bridge to residue E108 and hydrophobic contacts with F106, both in the H3 CDR of S309 (Figure 4E).

### Affinity of BA.2.86 for ACE2

We measured the affinity of BA.2.86 RBD for ACE2 using surface plasmon resonance (SPR). Biotinylated ACE2 was attached to a streptavidin-immobilized CM5 sensor chip (Cytiva) over which soluble RBD was flowed (Figures 4F and S2). Binding kinetics were close to ideal pseudo-first order. K_D_ for ACE2/BA.2.86 RBD was 8.3 nM, 2.2- and 1.7-fold higher than XBB.1.5 and Beta RBDs, respectively (the off-rate is notably slower for BA.2.86, Figure S2). The affinity of Beta RBD for ACE2 was itself 19-fold higher than we previously measured for ancestral Wuhan RBD (Figure 4G) and the increased affinity compared with XBB.1.5 may give BA.2.86 a transmission advantage against XBB.1.5-derived strains of SARS-CoV-2, which until recently dominated infections globally,^26^ although animal studies would be required to formally demonstrate this.

### Structural characterization of BA.2.86

**Figure 5:**
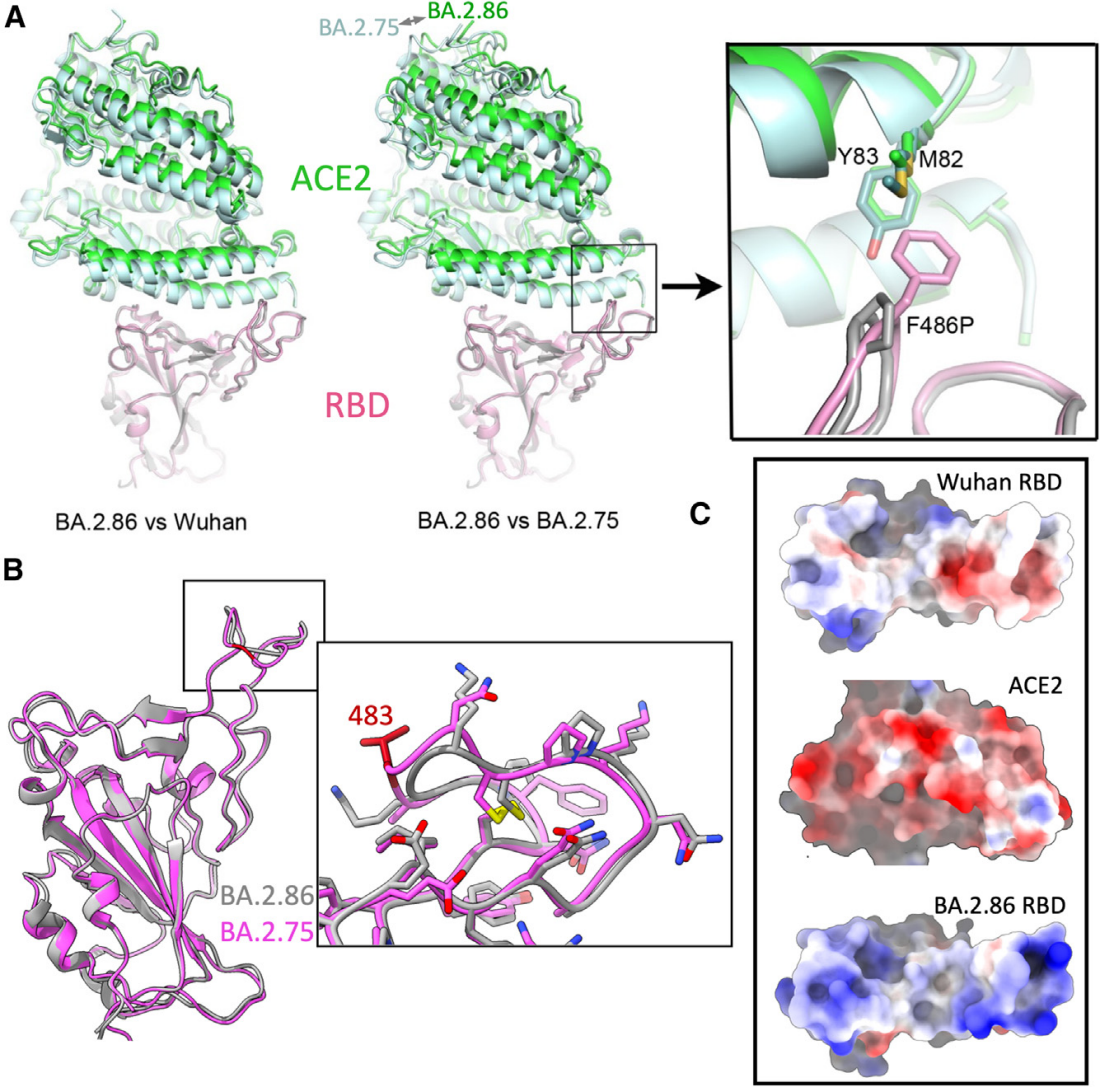
Structure of BA.2.86 ACE2 complex (A) Binding pose of ACE2 (green) to BA.2.86 RBD (gray) compared with binding pose of ACE2 (pale cyan) to Wuhan (left panel) and BA.2.75 (middle panel) RBD (pink). The right panel shows loss of direct contacts of ACE2 to residue 486 due to F486P mutation in BA.2.86 RBD. (B) Structural differences at the left shoulder between BA.2.86 (gray) and BA.2.75 (pink) RBDs due to V483 deletion in BA.2.86. (C) Electrostatic surfaces of the ACE2-RBD interface. See also Figures 4 and S2.

We determined the structure of the soluble trimeric S protein of BA.2.86 (in complex with XBB-7, see below). The RBD is rather mobile and not well ordered; however, it is possible to model and refine the structure of the RBD and it is clear that the numerous mutations and the deletion of residue 483 do not introduce major changes (RMSD compared with BA.2.75 RBD for 188 RBD Cα 0.56 Å, Figures 5A and 5B). The 483 deletion causes some changes in the loop but the major contact region with ACE2 (RBM) is not significantly changed, likely caused by the disulfide between residues 480 and 488 locking the structure in place (Figure 5B).

### Structure of ACE2 complexed with BA.2.86 trimeric spike

The cryo-EM structure of the complex was determined at a nominal resolution of 3.7 Å resolution. Previous analyses report one RBD bound,^30^ sometimes with partial occupancy of a second,^31^ and we see two RBDs in the up configuration with ACE2 attached, but neither of which is well ordered, consistent with flexibility of the RBDs. Nevertheless, using local refinement, we were able to model one ACE2/RBD complex using the BA.2.86 RBD structure from the XBB-7 complex described below and the ACE2 model from the complex with BA.2.75 RBD^24^ (Figure 5A). Given the limited resolution we were able to model only as rigid bodies. Comparing with the complex structure for the BA.2.75 (as a representative of earlier variants),^24^ we observe a small tilt of the ACE2 (Figure 5A). The effect is to move the C-terminal end of the first helix of ACE2, responsible for major interactions with the right shoulder of the RBD, slightly away from the RBD. This may be due to the loss of hydrophobic interactions due to the F486P mutation in the BA.2.86 RBD (Figure 5A) and is unlikely to contribute to the increased affinity for ACE2. This was confirmed by Yang et al.,^32^ who showed using surface plasmon resonance a notable reduction in ACE2 binding affinity for the JN.1 RBD.

However, by inspecting the electrostatic properties of the ACE binding surface, we speculate that the major driver for increased affinity, compared with the Wuhan strain, is electrostatic complementarity between BA.2.86 RBD and ACE2 (Figure 5C). Indeed, several of the mutations introduced into BA.2.86 have previously been identified as enhancing affinity, notably N440K, G446S, E484K, and Y505H.^33^^,^^34^ Finally, we speculate that the affinity for BA.2.86 RBD and ACE2 might be further enhanced by the flexibility of the RBDs, possibly improving the presentation of the ACE2 binding site in the context of virus-associated trimeric S protein.^35^

### Structures of XBB antibodies in complex with RBD and S trimer

**Figure 6:**
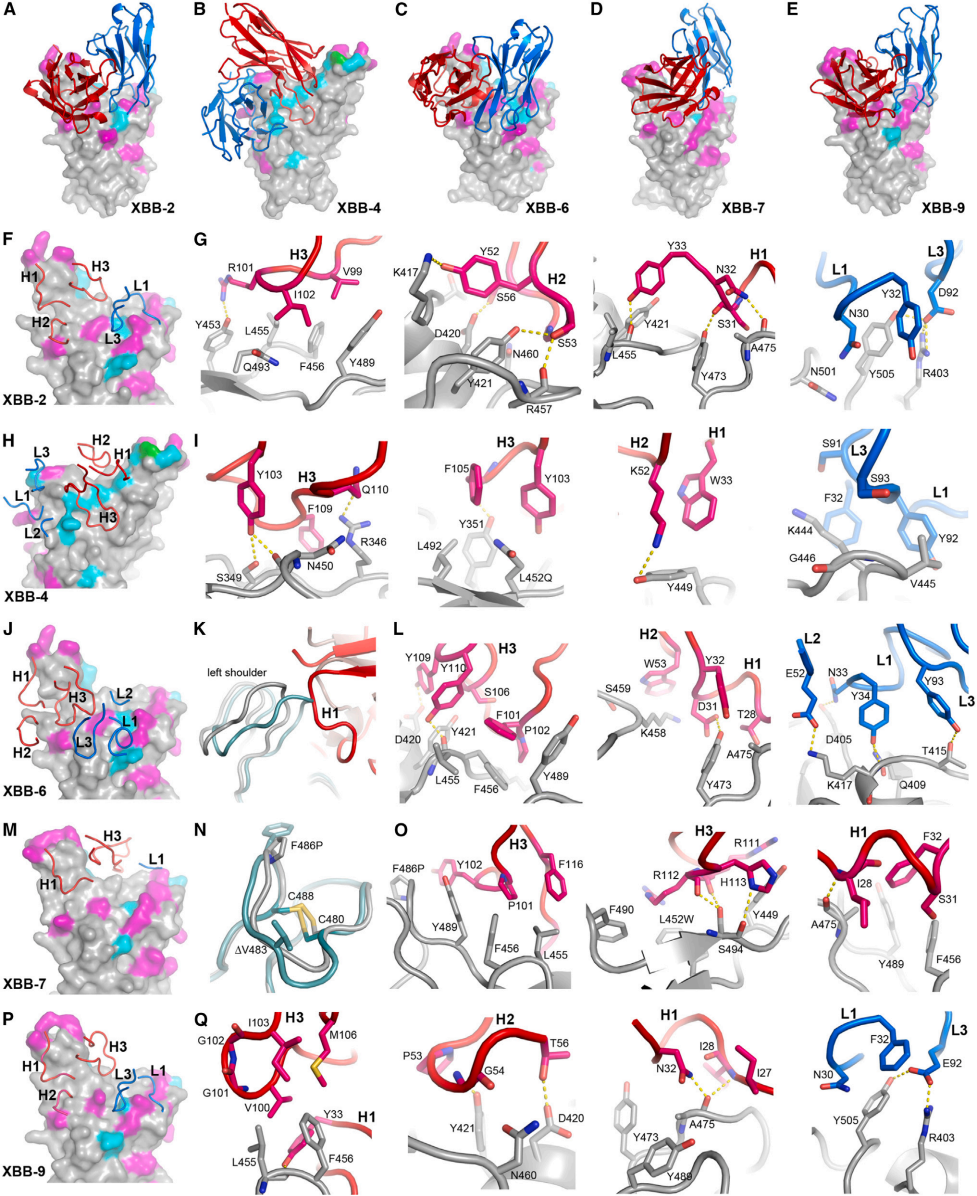
Structures of Delta-RBD/XBB-2, BA.2.12.1-RBD/XBB-4, delta-RBD/XBB-6, BA.2.86-RBD/XBB-7, and Delta-RBD/XBB-9 complexes (A–E) Binding pose of (A) XBB-2, (B) XBB-4, (C) XBB-6, (D) XBB-7, and (E) XBB-9 on the RBD, respectively. Only VH (red) and VL (blue) domains of the Fab are shown as ribbons for clarity. RBD is drawn as a gray surface representation with mutation sites common to XBB.1.5 and BA.2.86 highlighted in magenta, different or additional mutation sites in BA.2.86 in cyan. (F–I) (F) Positions of CDRs which have direct contacts (≤4.0 Å) with RBD, (G) details of Fab and RBD interactions for XBB-2, and (H and I) for XBB-4. The side chains of the RBD, Fab HC, and LC are shown as gray, red, and blue sticks, respectively. The yellow broken bonds represent hydrogen bonds or salt bridges. (J) Position of XBB-6 CDRs. (K) Structural changes of RBD left shoulder (gray) upon binding of XBB-6 (red) compared with the RBD (teal) bound with XBB-2 (brown). (L) Details of XBB-6 and RBD interactions. (M) Positions of XBB-7 CDRs that have direct contacts with the RBD. (N) Structural changes of BA.2.86 RBD (gray) due to deletion of V483 compared with XBB-2 bound Delta-RBD (teal). (O) Details of XBB-7 and RBD interactions. (P) Position of XBB-9 CDRs. (Q) Details of XBB-9 and RBD interactions. The drawing style and color scheme in (I), (L), (O), and (Q) are as in (G). See also Table S4.

The complex of XBB-2 Fab with Delta RBD and nanobody C1 was determined at 2.3 Å resolution by crystallography (Figures 6A, 6F, and 6G)*.* The antibody belongs to the IGHV3-53 variable gene family^36^ and binds in the pose characteristic of most of this family at the back of the RBD. While for many of the antibodies belonging to this public gene family neutralization was knocked out by variation in the RBD,^27^^,^^29^^,^^37^ XBB-2 has structural differences, notably in the light chain variable regions that allow it to effectively neutralize XBB. Despite contacting residues 455 and 456 in the RBD, this antibody can still neutralize viruses mutated at these residues, although at much reduced potency (Figure 4B). It is notable that 5/10 of the potent XBB mAbs belong to the public IGHV3-53/66 gene family and are likely to bind in a similar position to XBB-2. One of these, XBB-9, shows potent neutralization of all variants tested (see below).

The complex of XBB-4 Fab with the BA.2.12.1 S trimer was determined by Cryo-EM. The S trimer binds three Fabs; however, density for the RBDs and Fabs is poor. Local refinement including the SD1 and RBD domains of one spike chain and the bound Fab produced a density map at 3.4 Å resolution that enabled the model to be built. XBB-4 belongs to the IGHV3-15 gene family and is not sensitive to mutations of 455 and 456 at the back of the RBD but has much reduced neutralization of BA.2.86. XBB-4 binds in front of the RBD right shoulder with its long CDR-H3 extending from the right shoulder to the chest interacting with residues 346, 450, and 452 (Figures 6B, 6H, and 6I). R346T in XBB.1.5 and sub-variants and L452R in BA.4/5 have no significant effect on the neutralization (Figures 1A and 4B), therefore the N450D mutation is likely to be responsible for the resistance of BA.2.86 to neutralization. Both CDR-H1 and H2 make sole contact with Y449, one of the ACE2 footprint residues where mutation has not been observed so far. CDR-L3 interacts with residue V445. Mutation V445P in XBB.1.5 and its sub-variants does not affect the potency of XBB-4. V445 is mutated to a histidine in BA.2.86. Since the interaction is edge-on there is space to accommodate a larger histidine and the V445H change may also not affect neutralization.

The complex of XBB-6 Fab with Delta RBD and Beta 49 Fab was determined at 3.7 Å resolution by crystallography (Figures 6C and 6J–6L). XBB-6 belongs to the same gene family (IGHV3-9) as an anti-BA.1 antibody we identified earlier, Omi-42^9^, and binds in a very similar pose, also at the back of the RBD, in a similar orientation to XBB-2, with a modest 22° rotation but a shift of ∼7.5 Å toward the back of the left shoulder. This antibody uses RBD residues 455 and 456 for binding, with the interactions being much stronger than for XBB-2, and it is knocked out when these residues are mutated.

XBB-7 is more cross-reactive than XBB-2 and XBB-6, with little reduction in potency for RBDs bearing mutations at 455 and 456; however, its potency is reduced ∼8× against BA.2.86. We determined the structure of its Fab in complex with the BA.2.86 S trimer by cryo-EM at 3.6 Å resolution. This antibody belongs to the IGHV3-7 gene family and also binds at the back of the RBD in a similar orientation to XBB-2 but is shifted toward the right shoulder (Figures 6D and 6M–6O). Only three hypervariable loops form contacts with the RBD, H3, H1, and L1. The heavy chain CDR3 loop is unusually long (24 residues) and crosses over the top of the neck to the front of the RBD. RBD residue 456 contacts a proline from H3, leading to 4-fold reduction in potency for the F456L mutation in EG.5. However, the further adjacent mutation, L455F, seen in XBB.1.5.70, compensates for this, so there is little impact on potency.

The complex of XBB-9 Fab with Delta-RBD and an anti-Fab nanobody^38^ was determined at 4.0 Å resolution (Figures 6E, 6P, and 6Q). XBB-9 belongs to IGHV3-53 gene family and shows potent neutralization of all variants tested. As expected XBB-9 binds the RBD at a similar position and orientation to XBB-2 described above. CDR-H3 residues V100 and G101 contact L455 of the RBD, and residues I103 and M106 interact with F456 of the RBD. Interestingly, the F456L mutation in EG.5 increases the neutralization titer 3-fold compared with XBB.1.5, while the double mutations L455F and F456L in XBB.1.5.70 have no impact on neutralization potency. G101 and G102 at the tip of CDR-H3 may confer flexibility, allowing the H3 loop to adapt to changes at 455 and 456 of the RBD. XBB-9 neutralizes the JN.4 sub-variant of BA.2.86, which bears an A475V mutation. The carbonyl oxygen of A475 of the RBD makes bifurcated H-bonds to the N32 side chain and the I28 amide nitrogen of CDR-H1. The side chain of A475 also has close contacts with N32. The A475V change in JN.4 will clash with N32, but local structural changes at the interface of XBB-9 and JN.4 RBD are expected. CDR-H2 contacts N460 of the RBD and CDR-L1 and L3 interact with R403 and Y505 of the RBD; however, the N460K, R403K, and Y505H mutations have no impact on neutralization.

## Discussion

BA.2.86 harbors a large number of mutations (with the RBD alone having 24 substitutions and 1 deletion compared with Wuhan). Although the ΔV483 deletion has been seen before in SARS-CoV-2 sequences, the deletion of residues at the edge of the ACE2 binding surface of RBD has been very rare. Such a change might cause epistatic knock-on effects, reshaping the ACE2 interaction surface in a more profound way than simple amino acid substitution. However, we find that the structural changes are minor and localized; the presence of a disulfide bond close to ΔV483 appears to lock the loop, limiting the propagation of conformational change (Figure 5B). Despite the abundance of mutations within the ACE2 footprint (10 out of 25 residues mutated compared with Wuhan) we demonstrate that BA.2.86 has high affinity for ACE2 (a 2.2-fold increase compared with XBB.1.5). Structural analysis shows a minor change in binding mode for ACE2 compared with previous variants but suggests that the increase in affinity is due to improved electrostatic complementarity. Looking at a succession of earlier strains it appears that ACE2 affinity gains such as those seen with Alpha and Beta were lost when Omicron, carrying significant mutational burden, arose, with a drop in affinity accompanying the transition from Delta to Omicron. Binding was then recovered with the transition to BA.2.75 (Figure 4G). In contrast, it seems likely that BA.2.86 may already have sufficient ACE2 affinity to enable the rapid selection of further escape mutations, while the numerous changes in the RBD provide a shift in sequence and structure, also facilitating their structural accommodation.

Many mutations to BA.2 seen in BA.2.86 have also been acquired by other Omicron sublineages such as BA.4/5 and XBB, suggesting coevolution, presumably in response to shared immune selective pressures. However, the lack of intermediary viruses makes it likely that BA.2.86 has evolved sequentially, possibly in a chronically infected individual. Other variants such as Beta and Omicron first emerged in Southern Africa (which may be the origin of BA.2.86), where the high prevalence of HIV cases not on antiretroviral treatment can provide a substrate for chronic infections. Such infections have been observed for upward of a year, during which time considerable viral evolution has occurred.^19^^,^^20^^,^^39^ In some immunosuppressed individuals, the immune response may be sufficient to put pressure on the virus to evolve but insufficient to clear infection, leading to a long-term bootstrapping of viral and antibody evolution, until a virus is produced that is fit to escape into the immunocompetent environment.^20^ Indeed BA.2.86 can escape from all the potent mAbs we generated from BA.2-infected cases^29^ and appears to possess considerable resistance to BA.4 sera.^40^

The emergence of BA.2.86 raised concern that it may possess a more immune-evasive phenotype than currently circulating strains. Our results reported here and those from others,^41^^,^^42^ indicate that this is not the case. Considering that most people have received at least three doses of vaccine and a fourth dose has been administrated in some regions, we tested serum samples obtained 18 months after the third dose and 6 months after the fourth dose. Latest breakthrough infection samples are also crucial to assess the protection obtained by recent infection against BA.2.86, which can give an indication of a possible BA.2.86 reinfection. BA.2.86 is a variant derived from BA.2 and shares many similar mutations in S with BA.4/5, so it is worth testing the titers of serum samples obtained after BA.2 and BA.4/5 breakthrough infection to assess how much the multiple mutations in BA.2.86 contribute to its immune escape. A variety of vaccine and naturally infected sera show very similar neutralization profiles, with BA.2.86 being marginally easier to neutralize than XBB.1.5 and considerably easier to neutralize than EG.5. The donors of sera in the UK were largely multiply vaccinated health care workers and the highly related neutralization profiles that each group of sera displayed may result from similar imprinting of individual antibody responses; it is possible that sera from other areas, particularly where vaccine use has been less prevalent, may differ (Figure 2F).

We did not have access to many recently infected XBB cases, but Moderna have reported good neutralization of BA.2.86 following a boost with their most recent XBB.1.5-containing mRNA vaccine.^43^ In line with this, our analysis of a panel of potent anti-XBB mAbs produced from cases infected with contemporary strains, showed that only 1/10 of these (XBB-4) lost >90% neutralization ability against BA.2.86. Our structural analysis of complexes of 5 of the 10 potent mAbs (most of the remainder belong to the IGHV3-53/66 gene family and probably bind very similarly to one of those we analyzed) provides insight into this. We previously reported that anti-BA.2 mAbs^29^ showed a preference for epitopes on the front of the RBD. Presumably reflecting that this XBB variant and BA.2.86 have evolved independently to acquire numerous mutations on the front of the RBD (including R346T for XBB only, and G339D/H for XBB and BA.2.86, Figures 1B and 1C). In fact, there are more changes on the front of the RBD in BA.2.86 than XBB, and one of these changes, N450D, accounts for the loss of potency of XBB-4. These mutations likely account for the switch in focus of mAb binding in anti-XBB antibodies, which our structures show to be remarkably concentrated against epitopes on the back of the left shoulder of the RBD (8/10 mAbs). This region was widely used in potent responses made early in the pandemic, notably for IGHV3-53/66 antibodies, which also occur frequently (5/10) among the potent XBB neutralizers we isolate. Escape mutations in early variants generally knocked down the effectiveness of these antibodies, but it seems likely that further maturation of the responses has enabled some to recover potency. In addition, one antibody binding in this region uses an epitope uncommon in early responses (first identified for Omi-42, found following BA.1 infection^44^). Structural analyses of four mAbs binding at this back of the left shoulder region, also demonstrate direct interaction with residues 455/456 (Figure 6), which are mutated in the most recently circulating variants EG.5.1 (F456L) and XBB.1.5.70, which contain the so-called “flip mutations” L455F + F456L. In line with these structural results the activity of 7/10 potent XBB mAbs were knocked out or severely impaired when 455 and 456 were mutated. However, XBB-9, a VH3-53 mAb, despite binding in a very similar fashion, is not affected by 455, 456, or 475 mutations. The RBD-XBB-9 Fab structure suggests that this may be because G101 and G102 at the tip of XBB-9 CDR-H3 confer sufficient flexibility to accommodate these mutations (which simply change the size of hydrophobic side chains).

Thus, while an XBB.1.5-based mRNA vaccine should give some protection against BA.2.86 infection, our results suggest that this would focus responses to the RBD epitope containing residues 455 and 456, increasing pressure on BA.2.86 to acquire mutations in these residues such as the L455S mutation already seen in JN.1, leading to some degree of escape. JN.1 became the globally dominant strain at the end of 2023 and the majority of circulating variants at the moment are derived from it, as it accounts for 90.37% proportion within the past month (https://cov-spectrum.org/explore/World/AllSamples/Past1M/variants?nextcladePangoLineage=jn.1∗&). There are already 686 sequences submitted of JN.1 + F456L to GISAID (https://cov-spectrum.org/explore/World/AllSamples/AllTimes/variants?aaMutations=S%3Af456L&nextcladePangoLineage=jn.1∗&), which signifies the importance of the present results—it is important to learn from previously circulating variants to be prepared for upcoming JN.1 evolution. JN.1 significantly reduced effectiveness of vaccination or immunization by natural infection compared with BA.2.86 (Figure 2). The number of potent XBB antibodies is reduced from seven against BA.2.86 to three against JN.1, and all of the commercial mAbs remain ineffective against JN.1 (Figure 4).

In summary, we demonstrate here that BA.2.86 has not developed an extreme antibody escape phenotype but has sufficient ACE2 activity to be poised for further escape. Indeed, the acquisition of a single mutation L455S in JN.1 does lead to further antibody escape, probably underpinning the recent increase in JN.1. Based on the latest submitted sequencing results to GISAID, it seems that the 1-year-long XBB era has ended and JN.1 has become dominant globally, marking the start of evolution in the BA.2.86 era. One future direction may be indicated by the 686 recently submitted sequences with JN.1 + F456L, suggesting a possible future of “SLip” in the BA.2.86 background.

### Limitations of the study

The neutralization assays presented here are performed *in vitro* and may underestimate *in vivo* neutralization where antibody-dependent cell-mediated cytotoxicity and complement will be present.

## Consortia

The members of the OPTIC Consortium are Eleanor Barnes, Christopher Conlon, John Frater, Anni Jämsén, Katie Jeffery, Alexander Hargreaves, Priyanka Abraham, Miles Carroll, Stephanie Longlet, Melissa Govinder, Teresa Lambe, Callum Halstead, and Sofia Sampaio.

## STAR★Methods

### Key resources table

**Table:** 

REAGENT or RESOURCE	SOURCE	IDENTIFIER
**Antibodies**		

Fab	Dejnirattisai et al.^7^	N/A
IgG	Dejnirattisai et al.^7^ and Liu et al.^28^	N/A
Human anti-NP (mAb 206)	Dejnirattisai et al.^7^	N/A
Regeneron mAbs	AstraZeneca	Cat#REGN10933 and REGN10987
AstraZeneca mAbs	AstraZeneca	Cat#AZD1061, AZD8895 and AZD7442
Vir mAbs	Adagio	Cat#S309
Lilly mAbs	Adagio	Cat#Ly-CoV555, Ly-CoV16 and Ly-CoV1404
Adagio mAbs	Adagio	Cat#ADG10, ADG20 and ADG30
BA.2 antibodies	Dijokaite-Guraliuc et al.^29^	N/A
XBB antibodies	This paper	N/A

**Bacterial, virus strains, and yeast**		

DH5α bacteria	InVitrogen	Cat#18263012

**Biological samples**		

Serum from Pfizer-vaccinated individuals	University of Oxford	N/A
Plasma from SARS-CoV-2 patients (BA.2, BA.4/5, latest breakthrough infections)	John Radcliffe Hospital in Oxford UK, Sheffield	N/A

**Chemicals, peptides, and recombinant proteins**		

His-tagged SARS-CoV-2/XBB.1.5 RBD	This paper	N/A
His-tagged SARS-CoV-2/BA.2.86 RBD	This paper	N/A
His-tagged SARS-CoV-2/Beta RBD	Liu et al.^8^	N/A
Human ACE2-hIgG1Fc	Liu et al.^28^	N/A
Phosphate buffered saline tablets	Sigma-Aldrich	Cat#P4417
Dulbecco’s Modified Eagle Medium, high glucose	Sigma-Aldrich	Cat#D5796
Dulbecco’s Modified Eagle Medium, low glucose	Sigma-Aldrich	Cat#D6046
FreeStyle™ 293 Expression Medium	Gibco	Cat#12338018
L-Glutamine–Penicillin–Streptomycin solution	Sigma-Aldrich	Cat#G1146
GlutaMAX™ Supplement	Gibco	Cat#35050061
Opti-MEM™	Gibco	Cat#11058021
Fetal Bovine Serum	Gibco	Cat#12676029
Strep-Tactin®XT	IBA Lifesciences	Cat#2-1206-025
HEPES	Melford	Cat#34587-39108
LB broth	Fisher Scientific UK	Cat#51577-51656
Trypsin-EDTA	Gibco	Cat#2259288
TrypLE™ Express Enzyme	Gibco	Cat#12604013
L-Glutamine 200 mM (100X)	Gibco	Cat#2036885
Isopropyl β-*d*-1-thiogalactopyranoside	Meridian Bioscience	Cat#BIO-37036
Kanamycin	Melford	Cat#K22000
Ampicillin	Sigma-Aldrich	Cat#PHR2838
Agarose	Sigma-Aldrich	Cat#A2929
SYBR™ Safe DNA Gel Stain	Fisher Scientific UK	Cat#S33102
QIAprep Spin Miniprep Kit	Qiagen	Cat#27106X4
QIAquick® PCR & Gel Cleanup Kit	Qiagen	Cat#28704
Phusion™ High-Fidelity DNA Polymerase	Fisher Scientific UK	Cat#F530S
Bright-Glo™ Luciferase Assay System	Promega	Cat#E2620
HIV1 p24 ELISA Kit	Abcam	Cat#ab218268
NaCl	Sigma-Aldrich	Cat#S9888
Sensor Chip Protein A	Cytiva	Cat#29127555
Biotin CAPture Kit, Series S	Cytiva	CAT#28920234
HBS-EP+ Buffer 10×	Cytiva	Cat# BR100669
Regeneration Solution (glycine-HCl pH 1.7)	Cytiva	Cat# BR100838

**Deposited data**		

Crystal structures of: SARS-CoV-2 Delta-RBD/XBB-2/NbC1, SARS-CoV-2 Delta-RBD/XBB-6/Beta-49 and SARS-CoV-2 Delta RBD/XBB-9/Fab-Nb	This paper	PDB: 8QRG, PDB:8QRF, PDB:8R80
CryoEM structures of: SARS-CoV-2 BA.2.86-RBD/ACE2, BA.2.12.1-RBD/XBB-4 and BA.2.86-RBD/XBB-7 (all with local refinement)	This paper	PDB:8QSQ, EMDB:EMD-18639, PDB:8R8K, EMDB:EMD-19002, PDB:8QTD, EMDB:EMD-18649

**Experimental models: Cell lines**		

HEK293 cells	ATCC	Cat#CRL-3216
Expi293F™ Cells	Gibco	Cat#A14527
HEK293T/17 cells	ATCC	Cat#CRL-11268™
HEK293T cells	ATCC	Cat#CRL-11268
Vero CCL-81 cells	ATCC	Cat#CCL-81
VeroE6/TMPRSS2 cells	NIBSC	Ref. no. 100978

**Recombinant DNA**		

Vector: pHLsec	Aricescu et al.^45^	N/A
Vector: pNEO	Aricescu et al.^45^	N/A
Vector: pHLsec-SARS-CoV-2 spike of BA.2.86	This paper	N/A
Vector: pNEO-SARS-CoV-2 RBD of XBB.1.5	This paper	N/A
Vector: pNEO-SARS-CoV-2 RBD of BA.2.86	This paper	N/A
Vector: pNEO-SARS-CoV-2 RBD of Beta	Liu et al.^8^	N/A
Vector: pCMV-VSV-G	Stewart et al.^23^	Addgene plasmid # 8454
pHR-SIN-ACE2	Alain Townsend, Oxford	N/A
Vector: pcDNA-SARS-CoV-2 spike of BA.2 strain (T19I, Δ24–26, A27S, G142D, V213G, G339D, S371F, S373P, S375F, T376A, D405N, R408S, K417N, N440K, S477N, T478K, E484A, Q493R, Q498R, N501Y, Y505H, D614G, H655Y, N679K, P681H, N764K, D796Y, Q954H, N969K)	Nutalai et al.^9^	N/A
Vector: pcDNA-SARS-CoV-2 spike of BA.4/5 strain (T19I, Δ24–26, A27S, Δ69-70, G142D, V213G, G339D, S371F, S373P, S375F, T376A, D405N, R408S, K417N, N440K, L452R, S477N, T478K, E484A, F486V, Q498R, N501Y, Y505H, D614G, H655Y, N679K, P681H, N764K, D796Y, Q954H, N969K)	Tuekprakhon et al.^13^	N/A
Vector: pcDNA-SARS-CoV-2 spike of BN.1 strain (T19I, Δ24–26, A27S, G142D, K147E, W152R, F157L, I210V, V213G, G257S, D339H, R346T, K356T, S371F, S373P, S375F, T376A, D405N, R408S, K417N, N440K, G446S, N460K, S477N, T478K, E484A, F490S, Q498R, N501Y, Y505H, D614G, H655Y, N679K, P681H, N764K, D796Y, Q954H, N969K)	Dijokaite-Guraliuc et al.^29^	N/A
Vector: pcDNA-SARS-CoV-2 spike of XBB.1.5 strain (T19I, Δ24–26, A27S, V83A, G142D, Δ144, H146Q, Q183E, V213E, G252V, G339H, R346T, L368I, S371F, S373P, S375F, T376A, D405N, R408S, K417N, N440K, V445P, G446S, N460K, S477N, T478K, E484A, F486P, F490S, Q498R, N501Y, Y505H, D614G, H655Y, N679K, P681H, N764K, D796Y, Q954H, N969K)	This paper	N/A
Vector: pcDNA-SARS-CoV-2 spike of EG.5 strain (T19I, Δ24–26, A27S, V83A, G142D, Δ144, H146Q, Q183E, V213E, G252V, G339H, R346T, L368I, S371F, S373P, S375F, T376A, D405N, R408S, K417N, N440K, V445P, F456L, G446S, N460K, S477N, T478K, E484A, F486P, F490S, Q498R, N501Y, Y505H, D614G, H655Y, N679K, P681H, N764K, D796Y, Q954H, N969K)	This paper	N/A
Vector: pcDNA-SARS-CoV-2 spike of EG.5.1 strain (T19I, Δ24–26, A27S, Q52H, V83A, G142D, Δ144, H146Q, Q183E, V213E, G252V, G339H, R346T, L368I, S371F, S373P, S375F, T376A, D405N, R408S, K417N, N440K, V445P, F456L, G446S, N460K, S477N, T478K, E484A, F486P, F490S, Q498R, N501Y, Y505H, D614G, H655Y, N679K, P681H, N764K, D796Y, Q954H, N969K)	This paper	N/A
Vector: pcDNA-SARS-CoV-2 spike of XBB.1.5.70 strain (T19I, Δ24–26, A27S, V83A, G142D, Δ144, H146Q, Q183E, V213E, G252V, G339H, R346T, L368I, S371F, S373P, S375F, T376A, D405N, R408S, K417N, N440K, V445P, G446S, L455F, F456L, N460K, S477N, T478K, E484A, F486P, F490S, Q498R, N501Y, Y505H, D614G, H655Y, N679K, P681H, N764K, D796Y, Q954H, N969K)	This paper	N/A
Vector: pcDNA-SARS-CoV-2 spike of HK.3 strain (T19I, Δ24–26, A27S, Q52H, V83A, G142D, Δ144, H146Q, Q183E, V213E, G252V, G339H, R346T, L368I, S371F, S373P, S375F, T376A, D405N, R408S, K417N, N440K, V445P, G446S, L455F, F456L, N460K, S477N, T478K, E484A, F486P, F490S, Q498R, N501Y, Y505H, D614G, H655Y, N679K, P681H, N764K, D796Y, Q954H, N969K)	This paper	N/A
Vector: pcDNA-SARS-CoV-2 spike of BA.2.86 strain (ins16MPLF, T19I, R21T, L24del, P25del, P26del, A27S, S50L, H69del, V70del, V127F, G142D, Y144del, F157S, R158G, N211del, L212I, V213G, L216F, H245N, A264D, I332V, G339H, K356T, S371F, S373P, S375F, T376A, R403K, D405N, R408S, K417N, N440K, V445H, G446S, N450D, L452W, N460K, S477N, T478K, N481K, V483del, E484K, F486P, Q498R, N501Y, Y505H, E554K, A570V, D614G, P621S, H655Y, N679K, P681R, N764K, D796Y, S939F, Q954H, N969K, P1143L)	This paper	N/A
Vector: pcDNA-SARS-CoV-2 spike of JN.1 strain (ins16MPLF, T19I, R21T, L24del, P25del, P26del, A27S, S50L, H69del, V70del, V127F, G142D, Y144del, F157S, R158G, N211del, L212I, V213G, L216F, H245N, A264D, I332V, G339H, K356T, S371F, S373P, S375F, T376A, R403K, D405N, R408S, K417N, N440K, V445H, G446S, N450D, L452W, L455S, N460K, S477N, T478K, N481K, V483del, E484K, F486P, Q498R, N501Y, Y505H, E554K, A570V, D614G, P621S, H655Y, N679K, P681R, N764K, D796Y, S939F, Q954H, N969K, P1143L)	This paper	N/A
Vector: pcDNA-SARS-CoV-2 spike of JN.4 strain (ins16MPLF, T19I, R21T, L24del, P25del, P26del, A27S, S50L, H69del, V70del, V127F, G142D, Y144del, F157S, R158G, N211del, L212I, V213G, L216F, H245N, A264D, I332V, G339H, K356T, S371F, S373P, S375F, T376A, R403K, D405N, R408S, K417N, N440K, V445H, G446S, N450D, L452W, N460K, A475V, S477N, T478K, N481K, V483del, E484K, F486P, Q498R, N501Y, Y505H, E554K, A570V, D614G, P621S, H655Y, N679K, P681R, N764K, D796Y, S939F, Q954H, N969K, P1143L)	This paper	N/A
Vector: human IgG1 heavy chain	German Cancer Research Center, Heidelberg, Germany (H. Wardemann	N/A
Vector: human lambda light chain	German Cancer Research Center, Heidelberg, Germany (H. Wardemann	N/A
Vector: human kappa light chain	German Cancer Research Center, Heidelberg, Germany (H. Wardemann	N/A
Vector: Human Fab	University of Oxford	N/A
Vector: pJYDC1	Adgene	ID: 162458
TM149 BirA pDisplay	University of Oxford, NDM (C. Siebold)	N/A

**Software and algorithms**		

COOT	Emsley et al.^46^	https://www2.mrc-lmb.cam.ac.uk/personal/pemsley/coot/
Xia2-dials	Winter et al.^47^	https://xia2.github.io/parameters.html
Phaser	McCoy et al.^48^	https://www.ccp4.ac.uk/html/phaser.html
PHENIX	Liebschner et al.^49^	https://www.phenix-online.org/
PyMOL	Warren DeLano, Schrodinger	https://pymol.org/
CryoSPARC v4.1.2	Structura Biotechnology Inc.	https://cryosparc.com/
SerialEM (version 3.8.0 beta)	https://bio3d.colorado.edu/SerialEM/	N/A
EPU	Thermo Fisher	https://www.thermofisher.com/uk/en/home/electron-microscopy/products/software-em-3d-vis/epu-software.html
Prism 9.0	GraphPad	https://www.graphpad.com/scientific-software/prism/
IBM SPSS Software 27	IBM	https://www.ibm.com
Mabscape	Dejnirattisai et al.^7^ and Ginn et al.^50^	https://github.com/helenginn/mabscape, https://snapcraft.io/mabscape
Biacore T200 Evaluation Software 3.1	Cytiva	www.cytivalifesciences.com

**Other**		

X-ray data were collected at beamline I03, Diamond Light Source, under proposal ib27009 for COVID-19 rapid access	This paper	https://www.diamond.ac.uk/covid-19/for-scientists/rapid-access.html
Cryo-EM data were collected at OPIC, Division of Structural Biology, University of Oxford	This paper	https://www.opic.ox.ac.uk
TALON® Superflow Metal Affinity Resin	Clontech	Cat#635668
HiLoad® 16/600 Superdex® 200 pg	Cytiva	Cat#28-9893-35
Superdex 200 increase 10/300 GL column	Cytiva	Cat#28990944
HisTrap nickel HP 5-mL column	Cytiva	Cat#17524802
HiTrap Heparin HT 5-mL column	Cytiva	Cat#17040703
Amine Reactive Second-Generation (AR2G) Biosensors	Fortebio	Cat#18-5092
Octet RED96e	Sartorius	https://www.fortebio.com/products/label-free-bli-detection/8-channel-octet-systems
Buffer exchange system “QuixStand”	GE Healthcare	Cat#56-4107-78
Cartesian dispensing system	Genomic solutions	Cat#MIC4000
Hydra-96	Robbins Scientific	Cat#Hydra-96
96-well crystallization plate	Greiner bio-one	Cat#E20113NN
Crystallization Imaging System	Formulatrix	Cat#RI-1000
Sonics vibra-cell vcx500 sonicator	VWR	Cat#432-0137
Biacore T200	Cytiva	https://www.cytivalifesciences.com/en/us/shop/protein-analysis/spr-label-free-analysis/systems/biacore-t200-p-05644

### Resource availability

#### Lead contact

Resources, reagents and further information requirement should be forwarded to and will be responded by the lead contact, David I. Stuart (dave@strubi.ox.ac.uk).

#### Materials availability

Reagents generated in this study are available from the lead contact with a completed Materials Transfer Agreement.

#### Data and code availability

• Coordinates are deposited in the PDB and where appropriate EMDB: Delta-RBD/XBB-2/NbC1, PDB:8QRG. Delta-RBD/XBB-6/Beta-49, PDB:8QRF. Delta-RBD/XBB-9/Fab-Nb, PDB:8R80. BA.2.86-RBD/ACE2 local refinement, PDB:8QSQ, EMDB:EMD-18639. BA.2.12.1-RBD/XBB-4 local refinement, PDB:8R8K, EMDB:EMD-19002. BA.2.86-RBD/XBB-7 local refinement, PDB:8QTD, EMDB:EMD-18649.
• This paper does not report original code.
• Any additional information required to reanalyse the data reported in this paper is available from the lead contact upon request.

### Experimental model and subject details

#### Bacterial strains and cell culture

HEK293T (ATCC CRL-11268) cells were cultured in DMEM high glucose (Sigma-Aldrich) supplemented with 10% FBS, 1% 100X Mem Neaa (Gibco) and 1% 100X L-Glutamine (Gibco) at 37°C with 5% CO2. To express spike, RBD and ACE2, Expi293F cells (Thermo Fisher Scientific) were cultured in Expi293 Expression Medium (Thermo Fisher Scientific) at 37°C for transfection. Human mAbs were also expressed in HEK293T (ATCC CRL-11268) cells cultured in FreeStyle 293 Expression Medium (ThermoFisher, 12338018) at 37°C with 5% CO2. *E.coli DH5α* bacteria were used for transformation and large-scale preparation of plasmids. A single colony was picked and cultured in LB broth at 37 °C at 200 rpm in a shaker overnight.

#### *Sera from BA.2 infected cases,* study subjects

Following informed consent, healthcare workers with BA.2 infection were co-enrolled under the Sheffield Biobank study (STHObs) (18/YH/0441). All individuals had PCR-confirmed symptomatic disease and sequence confirmed BA.2 infection through national UKHSA sequencing data. A blood sample was taken following consent at least 12 days after PCR test confirmation. Clinical information including vaccination history, times between symptom onset and sampling and age of participant was captured for all individuals at the time of sampling (See Table S2).

#### Sera from BA.4/5 infected cases and breakthrough infections in the past 12M, study subjects

Following informed consent, individuals with omicron BA.4, BA.5, BA.2.73, BA.5.1, BA.5.2, XBB.1.5, BE.1, CH.1.1, CH.1.1.2 and BQ.1.1 were co-enrolled into one or more of the following three studies: the ISARIC/WHO Clinical Characterisation Protocol for Severe Emerging Infections [Oxford REC C, ref. 13/SC/0149], the “Innate and adaptive immunity against SARS-CoV-2 in healthcare worker family and household members” protocol (approved by the University of Oxford Central University Research Ethics Committee), or the Gastro-intestinal illness in Oxford: COVID sub study [Sheffield REC, ref. 16/YH/0247]. Diagnosis was confirmed through reporting of symptoms consistent with COVID-19, hospital presentation, and a test positive for SARS-CoV-2 using reverse transcriptase polymerase chain reaction (RT-PCR) from an upper respiratory tract (nose/throat) swab tested in accredited laboratories and lineage sequence confirmed through national reference laboratories in the United Kingdom. A blood sample was taken following consent at least 14 days after PCR test confirmation. Clinical information including severity of disease (mild, severe or critical infection according to recommendations from the World Health Organisation) and times between symptom onset and sampling and age of participant was captured for all individuals at the time of sampling (see Table S2).

#### Sera from vaccinees

V3 + 18M and V4 + 6M vaccine serum were obtained from volunteers who had received three doses of the Pfizer/BioNTech vaccine, Moderna vaccine or Oxford/AstraZeneca vaccine, and volunteers who had received three or four doses of Pfizer/BioNTech vaccine or Oxford/AstraZeneca vaccine before receiving a fourth (or fifth, 1 volunteer only) dose of Pfizer/BioNTech or Moderna bivalent vaccine (Table S2A). Vaccinees were Health Care Workers, based at Oxford University Hospitals NHS Foundation Trust, (previous infection history is shown in Table S2) and were enrolled in the OPTIC Study as part of the Oxford Translational Gastrointestinal Unit GI Biobank Study 16/YH/0247 [research ethics committee (REC) at Yorkshire & The Humber – Sheffield] which has been amended for this purpose on 15 March 2023. The study was conducted according to the principles of the Declaration of Helsinki (2008) and the International Conference on Harmonization (ICH) Good Clinical Practice (GCP) guidelines. Written informed consent was obtained for all participants enrolled in the study.

### Method details

#### Isolation of XBB.1.5 RBD-specific single B cells by FACS

XBB.1.5 RBD-specific single B cell sorting was performed as previously described.^7^ Briefly, 6 PBMCs of breakthrough infection (1 BA.4 infection, 1 BA.5.1 infection, 2 XBB.1.5 infection and 2 unknown infection) who were infected by BA.4, BA.5.1 or XBB.1.5 were stained with LIVE/DEAD Fixable Aqua dye (Invitrogen). Cells were then incubated with CD3-FITC, CD14-FITC, CD16-FITC, CD56-FITC, IgM-FITC, IgA-FITC, IgD-FITC, IgG-BV786 and CD19-BUV395, along with Strep-MAB-DY549 to stain the twin strep tag of the XBB.1.5 SD1-RBD protein, and anti-His-APC to stain the 6 × His tag of the XBB.1.5 RBD. IgG+ memory B cells were gated as CD19^+^, IgG+, CD3^−^, CD14^−^, CD56^−^, CD16^−^, IgM-, IgA- and IgD-, and XBB.1.5 SD1-RBD and XBB.1.5 RBD double-positive was further selected, and single cells were sorted into 96-well PCR plates with 10 μL of catching buffer (Tris, Nuclease-free-H2O and RNase inhibitor). Plates were briefly centrifuged at 2000ⅹg for 1 min and left on dry ice before being stored at −80°C.

#### Antigenic mapping

Antigenic mapping was carried out using Mabscape.^7^^,^^13^^,^^50^ In short, coronavirus variants were assigned coordinates (initially chosen randomly) whereby the distance between two points indicates the base drop in neutralization titer. Each serum was assigned a strength parameter which provided a scalar offset to the logarithm of the neutralization titer. These starting parameters were refined to match predicted neutralization titers to observed values. This was repeated and the final map was the average of superimposed positions from 20 separate runs. The positions of the variants were plotted for display. Previously the 3D coordinates were refined. For these data we found that the match of predicted and observed titers was almost equally good for a 2D model, and so the simpler 2D model is presented here.

#### Cloning and expression of XBB.1.5 RBD-specific human mAbs

XBB.1.5 RBD-specific human mAbs were cloned and expressed as described previously.^7^ Briefly, genes for Ig IGHV, Ig Vκ and Ig Vλ were recovered from positive wells by RT-PCR. Genes encoding Ig IGHV, Ig Vκ and Ig Vλ were then amplified using Nested-PCR by a cocktail of primers specific to human IgG. PCR products of HC and LCs were ligated into the expression vectors of human IgG1 or immunoglobulin κ-chain or λ-chain by Gibson assembly.^51^ For mAb expression, plasmids encoding HCs and LCs were co-transfected into a HEK293T cell line by PEI-transfection, and supernatants containing mAbs were collected and filtered 4–5 days after transfection, and the supernatants were purified.

#### Pseudovirus plasmid construction and lentiviral particles production

Pseudotyped lentivirus expressing SARS-CoV-2 S proteins from BA.1, BA.2, BA.4/5, and XBB.1 were constructed as described previously.^9^^,^^13^^,^^23^^,^^40^ The same method was used to construct XBB.1.5 by introducing F486P mutation into XBB.1, EG.5 by introducing F456L mutation into XBB.1.5, EG.5.1 by introducing Q52H into EG.1, XBB.1.5.70 by adding L455F into EG.5, and HK.3 by introducing Q52H into XBB.1.5.70. The plasmid to create BA.2.86 PV was custom synthesized by Integrated DNA Technologies based on the wild-type SARS-CoV-2 BA.2.86 (EPI_ISL_18110065) and cloned into pcDNA3.1 plasmid.^22^ This plasmid carries S gene and was used for generating pseudoviral particles together with the lentiviral packaging vector and transfer vector encoding luciferase reporter. A BA.2.86 plasmid containing the following mutations was produced: ins16MPLF, T19I, R21T, L24del, P25del, P26del, A27S, S50L, H69del, V70del, V127F, G142D, Y144del, F157S, R158G, N211del, L212I, V213G, L216F, H245N, A264D, I332V, G339H, K356T, S371F, S373P, S375F, T376A, R403K, D405N, R408S, K417N, N440K, V445H, G446S, N450D, L452W, N460K, S477N, T478K, N481K, V483del, E484K, F486P, Q498R, N501Y, Y505H, E554K, A570V, D614G, P621S, H655Y, I670V, N679K, P681R, N764K, D796Y, S939F, Q954H, N969K, P1143L. JN.1 was created by introducing L455S into BA.2.86 and JN.4 by introducing A475V into BA.2.86. All the constructs were sequence confirmed.

#### Pseudoviral neutralization test

The pseudoviral neutralization test has been described previously.^8^ Briefly, 4-fold serial diluted mAbs were incubated with pseudoviral particles at 37°C, 5% CO_2_ for 1 h. Stable HEK293T/17 cells expressing human ACE2 were then added to the mixture at 1.5 × 10^4^ cells/well. 48 h post infection, culture supernatants were removed and 50 μL of 1:2 Bright-Glo TM Luciferase assay system (Promega, USA) in 1 × PBS was added to each well. The reaction was incubated at room temperature for 5 min and firefly luciferase activity was measured using CLARIOstar (BMG Labtech, Ortenberg, Germany). The percentage neutralization was calculated relative to the control. Probit analysis was used to estimate the dilution that inhibited half maximum pseudotyped lentivirus infection (PVNT50).

To determine the neutralizing activity of convalescent plasma/serum samples or vaccine sera, 3-fold serial dilutions of each sample were incubated with pseudoviral particles for 1 h and the same strategy as mAb was applied.

#### Construction of trimeric spike of SARS-CoV-2 BA.2.86

Expression plasmid of BA.2.86 spike was constructed encoding for human codon-optimized sequences from wild-type SARS-CoV-2 (MN908947) and BA.2.86 (EPI_ISL_18110065). Fragments were cloned in pHLsec vectors^45^ downstream of the chicken β-actin/rabbit β-globin hybrid promoter and followed by a T4 fibritin trimerization domain, an HRV 3C cleavage site, a His-8 tag and a Twin-Strep-tag at the C terminus as previously reported by Wrapp et al., 2020.^34^ Mutations coding for stabilizing proline residues and to eliminate putative furin cleavage sites were inserted in BA.2.86 sequence as follows: RRAR > GSAS (aa 682–685) and KV > PP (aa 986–987). Spike includes following mutations: ins16MPLF, T19I, R21T, L24del, P25del, P26del, A27S, S50L, H69del, V70del, V127F, G142D, Y144del, F157S, R158G, N211del, L212I, V213G, L216F, H245N, A264D, I332V, G339H, K356T, S371F, S373P, S375F, T376A, R403K, D405N, R408S, K417N, N440K, V445H, G446S, N450D, L452W, N460K, S477N, T478K, N481K, V483del, E484K, F486P, Q498R, N501Y, Y505H, E554K, A570V, D614G, P621S, H655Y, I670V, N679K, P681R, N764K, D796Y, S939F, Q954H, N969K, P1143L. Spike fragments were custom synthesized by Integrated DNA Technologies and cloned into pHLsec vector as previously described.^7^^,^^25^^,^^26^ Spike sequence was verified by Sanger sequencing.

#### Cloning of RBDs

Gene fragment encoding RBD was ordered from Integrated DNA Technologies. This gene fragment comprises a 5′ tag (5′- GTTGCGTAGCTGAAACCGGT-3′), DNA sequence encoding a 6 × His tag, human codon-optimized DNA sequence of RBD BA.2.86 (332-526aa) and a 3′ tag (5′- AACAGCACCTCAAGGGTACC-3′). Vector pHR-CMV-TetO2_IRES-EmGFP was cut with restriction enzymes AgeI and KpnI and was assembled with the gene fragment using In-Fusion cloning. *E.coli* DH5α bacteria were used for transformation of plasmids and single colonies were picked and cultured in LB broth. Sequence of extracted plasmid was confirmed by Sanger sequencing.

Constructs of other RBD proteins used in this paper are as previously described^7^^,^^25^^,^^26^

#### Production of proteins

Protein expression and purification were as reported previously.^7^^,^^26^ Briefly, Twin-strep tagged BA.2.86 spike was transiently transfected in HEK293T cells and purified with Strep-Tactin XT resin (IBA lifesciences). Purified protein was validated by SDS-PAGE and concentrated using a 100 kDa Amicon Centrifugal Filter.

Plasmid encoding RBD was transiently transfected into Expi293F cells. Four days after transfection, the conditioned medium was harvested, filtered and buffer-exchanged using QuixStand benchtop system (Amersham Biosciences). The sample was purified with a 5 mL HisTrap nickel column (Cytiva) and further polished using a Superdex 75 HiLoad 16/60 gel filtration column (Cytiva). SDS-PAGE was used to validate the protein and protein was concentrated using a 10 kDa Amicon Centrifugal Filter.

#### Surface plasmon resonance

All SPR experiments were carried out at 37°C on a Biacore T200 system using HBS-EP+ buffer (Cytiva). Biotinylated ACE2 (19-615aa)-Avi^52^ was captured using a streptavidin-immobilised CM5 sensor chip (Cytiva). The final capture level was 100 RU. A 2-fold serial dilution of seven concentrations of different RBDs starting at 320 nM was used to pass over the immobilized ACE2-biotin. All runs were reference and blank subtracted and normalised against the baseline RU levels prior to injection of the RBD analytes. The SPR data were fitted using the curve fitting “one site – specific binding” equation in GraphPad Prism (version 10.0.3) to derive the equilibrium dissociation constant (KD) values. The R2 of all fits were >0.95.

#### IgG mAbs and fabs production

AstraZeneca and Regeneron antibodies were provided by AstraZeneca, Vir, Lilly and Adagio antibodies were provided by Adagio, LY-CoV1404 was provided by LifeArc. For the in-house antibodies, heavy and light chains of the indicated antibodies were transiently transfected into 293T cells and antibody purified from supernatant on protein A as previously described.^9^ Fabs were digested from purified IgGs with papain using a Pierce Fab Preparation Kit (Thermo Fisher), following the manufacturer’s protocol.

#### Crystallization, X-Ray data collection and structure determination

Delta-RBD was deglycosylated with Endoglycosidase F1 before used for crystallization. Ternary complexes of Delta-RBD/XBB-2/NbC1, Delta-RBD/XBB-6/Beta49 and Delta-RBD/XBB-9/Anti-Fab Nanobody were made by mixing proteins together in a 1:1:1 M ratio, with a final concentration of 11 mg mL^−1^ and 7 mg mL^−1^, separately. Screening of crystals at 20°C was set up in Crystalquick 96-well X plates (Greiner Bio-One) with a Cartesian Robot using the nanoliter sitting-drop vapor-diffusion method, with 100 nL of protein plus 100 nL of reservoir in each drop, as previously described.^53^ Crystals of Delta-RBD/XBB-2/NbC1 were obtained from Hampton Research PEGRx condition 2–27, containing 2% (v/v) 1,4-Dioxane, 0.1 M Tris pH 8.0 and 15% (w/v) PEG 3350. Crystals of Delta-RBD/XBB-6/Beta49 were obtained from Hampton Research PEGRx condition 1–40, containing 0.1 M citric acid pH 3.5 and 28% (w/v) PEG 8000. Crystals of Delta-RBD/XBB-9/anti-Fab nanobody were grown in solution containing 0.2 M Calcium acetate hydrate, 0.1 M sodium cacodylate pH 6.5 and 40% (v/v) PEG 300.

Crystals were mounted in loops and dipped in solution containing 25% glycerol and 75% mother liquor for a second before being frozen in liquid nitrogen. Diffraction data were collected at 100 K at beamline I03 of Diamond Light Source, UK, using the automated queue system that allows unattended automated data collection (https://www.diamond.ac.uk/Instruments/Mx/I03/I03-Manual/Unattended-Data-Collections.html). 3600 diffraction images of 0.1° each were collected for each dataset. Data were automatically processed with Xia2-dials.^47^^,^^54^ Each of the structures was determined using molecular replacement with Phaser^48^ and a model of our previously determined RBD/Fab structures that has maximum sequence identity with the current structure.^7^^,^^8^^,^^9^^,^^29^ Model rebuilding is done with COOT^46^ and refinement with Phenix.^49^ Due to the low resolution of Delta-RBD/XBB-6/Beta-49 and Delta-RBD/XBB-9/anti-Fab nanobody datasets, reference model restraints were applied in the refinement.

Data collection and structure refinement statistics are given in Table S4. Structural comparisons used SHP^55^ and figures were prepared with PyMOL (The PyMOL Molecular Graphics System, Version 1.2r3pre, Schrödinger, LLC).

#### Cryo-EM grid preparation

3 μL aliquots of trimeric S at ∼1.2 μM with fab or ACE2 in 6-fold molar excess of S (2-fold molar excess of binding sites) were prepared, aspirated and almost immediately applied to a freshly glow-discharged Cflat 2/1–200 mesh holey grid (Protochips, supplied by Molecular Dimensions) at high intensity, 20 s, Plasma Cleaner PDC-002-CE, Harrick Plasma. Excess liquid was removed by blotting for 5 s with a force of −1 using vitrobot filter paper (grade 595, Ted Pella Inc.) at 4.5°C, 100% reported humidity prior plunge freezing into liquid ethane using a Vitrobot Mark IV (Thermo Fisher).

#### Cryo-EM data collection and analysis, generic

Data were collected in EER format using EPU on a 300 kV Titan Krios G3i microscope equipped with a Falcon-IVi detector and selectrisX energy filter using EPU software (Thermofisher) and employing 50 μm C2, 100 μm objective apertures (Table S1). A total dose of 50 e/Å^2^ was applied and movies recorded at 165 kX magnification, corresponding to a calibrated pixel size 0.7303 Å/pix and multiple shots per hole (9–10) were recorded. Movies were 4-times binned and pre-processed (motion, CTF correction and blob particle picking) on the fly using the cryoSPARCv4.3.1 live.^56^ Movies were ‘cleaned’ using the live interface based on CTF estimation, defocus estimates, total motion and ice thickness. Subsequent analysis used CryoSPARC.^56^

Details are provided for all three structures in Table S4.

#### Cryo-EM analysis, BA.2.86 spike with XBB-7 fab

A total of 9069 movies were processed from which 524,958 particles were initially picked, which were filtered by 2D classification with 250 classes and a batch size of 200. Good classes, bearing clear secondary structure, were then selected, corresponding to 168,720 particles showing a variety of orientations. Particles were then aligned using a map generated from ab initio processing of this good subset before heterogeneous refinement into three classes. From heterogeneous refinement, 147389 particles fell into a class that was well aligned and resolved. This set was then un-binned and refined. Although clearly only one ‘upwards’ RBD was decorated with fab, and the non-uniform refined structure was globally high resolution (2.6 Å GSFSC 0.143 reported), density for the RBDs and RBD/fab was extremely poor. To improve on this a series of 3D classifications and local refinements were trialled to get a better view of Fab/RBD interface. It was found that a small number of particles were clearly decorated with two fabs (ca. 1.5%), some with one relatively better resolved Fab and one potentially upwards RBD decorated with fab (33%) but the better resolved had more clearly just one Fab bound (ca. 40%).

For the best strategy, 3D classification without alignment into eight classes with a focused mask around the top, i.e., S1/XBB-7 portion of the spike was performed. Subsequent refinement of the separated particles did not help resolve the RBDs/XBB-7 sufficiently for model building. The best subset, corresponding to 75021 particles was, therefore, locally refined twice, focusing on the best resolved XBB-7/RBD, resulting in a final reconstruction to 3.6 Å reported resolution (GSFSC, 0.143 CryoSPARC^56^).

#### Cryo-EM analysis, BA.2.12.1 spike with XBB-4

A total of 6616 exposures were collected and pre-processed on-the-fly using cryoSPARC live. A total of 874,792 picked particles were classified into 250 classes, from which 21 bearing clear secondary structure and a variety of orientations were selected (164,337 particles). These 4xbinned particles were then aligned in 3D before undergoing heterogeneous refinement with three reference volumes (one of which seemed to be spike decorated with fabs with RBDs in a variety of poses) generated *ab intio* from a particle subset. The 114,896 particles belonging to a class corresponding to convincing Spike were refined, extracted to the final box size and then further refined to 2.8 Å resolution. Since the fab/RBD interface was poorly resolved, this particle set was then further classified using various masks focusing on either one RBD/fab, the two closest RBDs/fabs, and the crown of the complex (three RBDs, 3 fabs) generated using a heavily filtered refined map. For each trial, the classes were selected and then locally refined again trialling a variety of masks. The best strategy was found to be alignment-free classification focusing on the crown of the complex, selection of the most populated class (61,333 particles) and refinement of this class (2.9 Å resolution) followed by two rounds of local refinement focusing on the best resolved of the RBD/fabs (respective resolutions/bfactors: 3.43 Å, −73.1 then 3.41 Å, −69) with a stricter shift search on the second round (2 Å to 1 Å, 3 deg rotation search). This locally refined map was sufficient to model the fab/RBD interface.

#### Cryo-EM analysis, BA.2.86 spike with ACE2

9818 movies were collected and particles 504319 picked initially. These were sorted by 2D classification, where 128,404 particles, in classes showing secondary structural detail and a variety of views were selected. Initial 3D processing via heterogeneous refinement using three ab-initio generated volumes followed by non-uniform refinement^57^ (103377) again showed poor density for the RBDs and RBD/ACE2 region.

B-factor blurring this initial map as above suggested two decorated RBDs. Various strategies were then trialled to locate a subset of well aligned particles. For the best strategy, the aligned ‘good’ particle set was 3D classified without alignment into eight classes. It was observed that classes showed a continuum of RBD poses in the upwards position, where the more separated the two ACE2-bearing RBDs were separated, the better resolved (with half of the classes having a better resolved RBD/ACE2-1; the other half a better resolved RBD/ACE2-2. Following class inspection, the best class in terms of ACE2/RBD resolution was then selected (41051), refined and then classified again, this time with a focused mask around the relatively well resolved RBD/ACE2 for this particle subset, again without alignment into five classes. The class bearing the best resolved RBD/ACE2 (15,883 particles) was then locally refined focusing again at this interface, resulting in a final map with a CryoSPARC^56^ reported GSFSC resolution of 3.7 Å.

### Quantification and statistical analysis

Statistical analyses are reported in the results and figure legends. Neutralization was measured on pseudovirus. The percentage reduction was calculated and IC_50_ determined using the probit program from the SPSS package. The Wilcoxon matched-pairs signed rank test was used for the analysis and two-tailed *p* values were calculated on geometric mean values.

## Supplemental information

Document S1. Figures S1 and S2 and Tables S1–S4

Document S2. Article plus supplemental information
