# Supplementary material for: A structure-function analysis shows SARS-CoV-2 BA.2.86 balances antibody escape and ACE2 affinity
Source: Cell Rep Med. 2024 May 8;5(5):101553. doi: 10.1016/j.xcrm.2024.101553 (PMC11148769; doi:10.1016/j.xcrm.2024.101553)
Supplement: Document S1. Figures S1 and S2 and Tables S1–S4 [file mmc1.pdf]

Cell Reports Medicine, Volume *5*

Supplemental information

A structure-function analysis shows SARS-CoV-2 BA.2.86 balances antibody escape and ACE2 affinity

Chang Liu, Daming Zhou, Aiste Dijokaite-Guraliuc, Piyada Supasa, Helen M.E. Duyvesteyn, Helen M. Ginn, Muneeswaran Selvaraj, Alexander J. Mentzer, Raksha Das, Thushan I. de Silva, Thomas G. Ritter, Megan Plowright, Thomas A.H. Newman, Lizzie Stafford, Barbara Kronsteiner, Nigel Temperton, Yuan Lui, Martin Fellermeyer, Philip Goulder, Paul Klenerman, Susanna J. Dunachie, Michael I. Barton, Mikhail A. Kutuzov, Omer Dushek, OPTIC Consortium, Elizabeth E. Fry, Juthathip Mongkolsapaya, Jingshan Ren, David I. Stuart, and Gavin R. Screaton

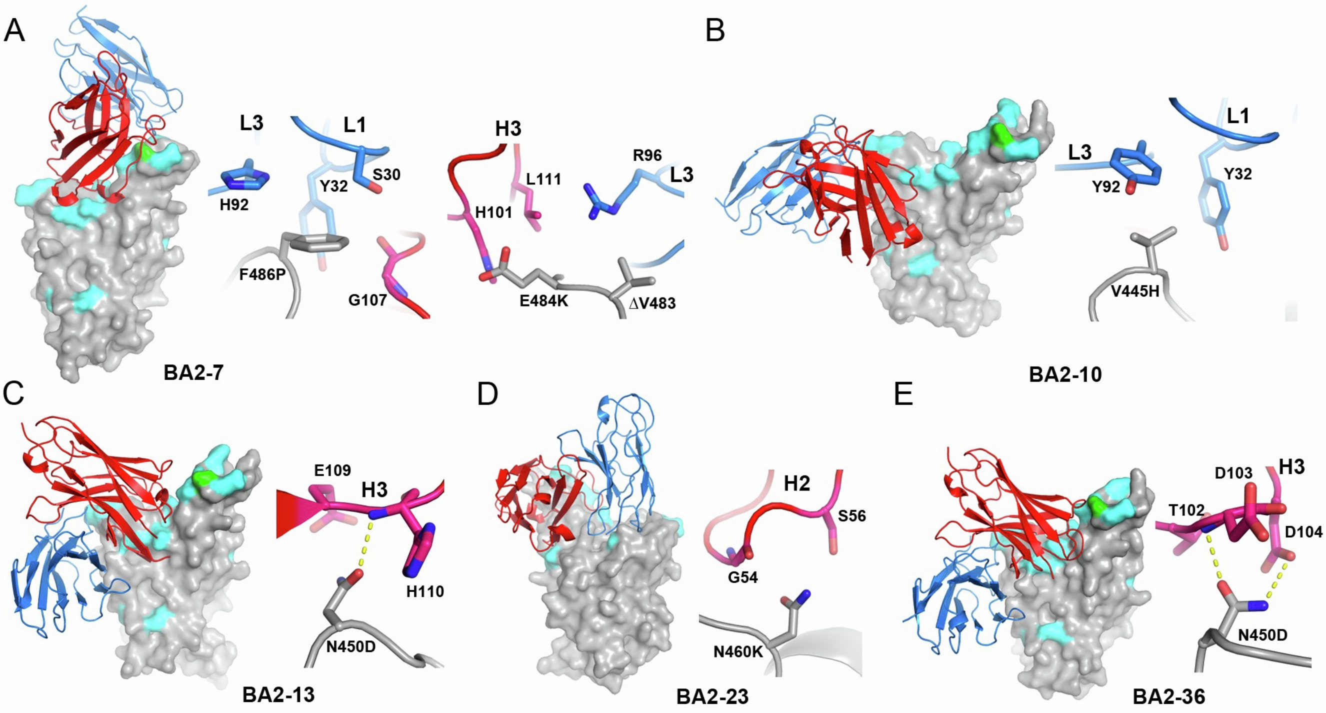

Figure S1. BA.2.86 mutations which knock out structurally known BA.2 mAbs.

(A)-(E) Binding mode and interactions to residues which are mutated in BA.2.86 for BA2-7, BA2-10, BA2-13, BA2-23 and BA2-36, respectively. Fab heavy chains are shown in red, light chains in blue. RBD shown as grey surface with BA.2.86 mutation site in cyan. Side chains are shown as grey, red and blue sticks for RBD, Fab heavy chain and light chain, respectively. Related to Figure 3.

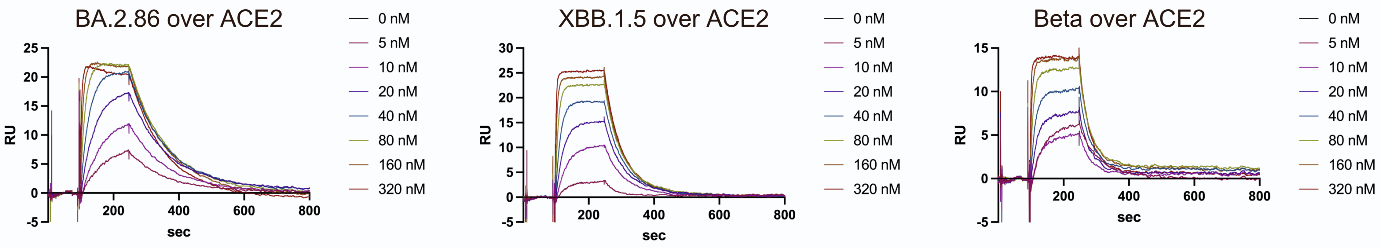

Figure S2. Sensorgrams related to kinetics data presented in Figure 4F

Mutation	Total number of sequences (2020-01-06 to 2023-12-26)	Variants in which mutation been observed	Number of sequences from BA.2.86 lineage
**Ins16MPLF**	24317	JN.1 (52.43%), JN.1.1 (16.53%), BA.2.86.1 (12.30%)	24098
**R21T**	39389	JN.1 (36.34%), AY.70 (14.06%), JN.1.1 (11.16%)	28003
**S50L**	30254	JN.1 (47.07%), JN.1.1 (14.62%), BA.2.86.1 (13.63%)	28939
H69del	6204108	B.1.1.7 (18.29%), BA.1.1 (16.30%), BA.1 (6.93%)	28514
V70del	6184237	B.1.1.7 (18.27%), BA.1.1 (16.32%), BA.1 (6.94%)	28351
**V127F**	33245	JN.1 (47.81%), JN.1.1 (14.06%), BA.2.86.1 (13.07%)	31270
Y144del	4668764	B.1.1.7 (23.76%), BA.1.1 (21.16%), BA.1 (9.06%)	30289
**F157S**	50923	JN.1 (30.29%), B.1.637 (29.96%), JN.1.1 (8.92%)	30279
R158G	4169523	AY.4 (20.04%), AY.103 (7.18%), AY.43 (6.91%)	30346
N211del	2152716	BA.1.1 (40.98%), BA.1 (17.35%), BA.1.17.2 (8.69%)	30207
L212I	2119227	BA.1.1 (41.15%), BA.1 (17.44%), BA.1.17.2 (8.78%)	29943
**L216F**	41851	JN.1 (36.85%), JN.1.1 (10.85%), BA.2.86.1 (10.04%)	30281
**H245N**	44428	JN.1 (34.96%), BA.2.3.20 (11.07%), JN.1.1 (10.28%)	30479
**A264D**	30493	JN.1 (50.26%), JN.1.1 (14.79%), BA.2.86.1 (13.57%)	29973
**I332V**	30628	JN.1 (50.32%), JN.1.1 (14.70%), BA.2.86.1 (13.64%)	30181
G339H	1150488	XBB.1.5 (16.19%), XBB.1.16 (3.00%), EG.5.1.1 (2.46%)	30097
K356T	115645	BN.1.3 (15.66%), JN.1 (13.24%), BN.1.2 (8.35%)	29877
**R403K**	32827	JN.1 (42.07%), JN.1.1 (12.83%), BA.2.86.1 (10.49%)	26477
**V445H**	29829	JN.1 (50.43%), JN.1.1 (15.01%), BA.2.86.1 (13.76%)	29506
G446S	2924202	BA.1.1 (27.38%), BA.1 (10.05%), XBB.1.5 (6.15%)	29582
N450D	67246	JN.1 (22.43%), BF.14 (11.84%), BA.2.3.20 (7.22%)	29576
**L452W**	29916	JN.1 (50.04%), JN.1.1 (14.89%), BA.2.86.1 (13.62%)	29320
N460K	1621814	XBB.1.5 (11.20%), BQ.1.1 (9.99%), BQ.1 (3.04%)	29630
**N481K**	33058	JN.1 (45.49%), JN.1.1 (13.33%), BA.2.86.1 (12.11%)	29169
**V483del**	25992	JN.1 (43.76%), JN.1.1 (14.87%), BA.2.86.1 (12.07%)	23077
E484K	294161	P.1 (26.21%), B.1.351 (11.15%), B.1.526 (8.51%)	27463
F486P	912141	XBB.1.5 (20.23%), XBB.1.16 (3.59%), HV.1 (3.12%)	29514
Q493R reversion	10701421	B.1.1.7 (10.62%), AY.4 (8.09%), BA.5.2 (3.05%)	30005
**E554K**	43945	JN.1 (35.73%), JN.1.1 (10.51%), BA.2.86.1 (9.76%)	30873
**A570V**	33638	JN.1 (46.83%), JN.1.1 (13.76%), BA.2.86.1 (12.78%)	30966
**P621S**	40009	JN.1 (39.14%), JN.1.1 (11.54%), BA.2.86.1 (10.58%)	30732
I670V	4741	BA.4.1.1 (63.13%), B.1.1.7 (4.47%), AY.4 (2.66%)	9
P681R	4527158	AY.4 (19.49%), AY.103 (7.10%), B.1.617.2 (6.77%)	30580
S939F	61793	JN.1 (25.00%), JN.1.1 (7.38%), BA.2.86.1 (6.74%)	30252
**P1143L**	35514	JN.1 (44.27%), JN.1.1 (12.99%), BA.2.86.1 (12.03%)	30848

Table S1. Mutations in BA.2.86 compared to BA.2 and their prevalence in previous variants. Related to Figure 1.

A

		V3+18M	V4+6M	BA.2 Infection	BA.4/5 Infection	Latest Infection
**Participants**						
Female		12	16	16	6	16
Male		5	7	3	4	3
**Median age (Y)**		36 (Range 25-68)	43 (Range 24-65)	45 (Range 22-57)	43 (Range 20-94)	
**Vaccine History**						
First dose						
	Pfizer/BioNtech	13	17	16	7	
	Oxford/AstraZeneca	3	6	3	2	
	Moderna	1	0			
Second dose						
	Pfizer/BioNtech	13	17	16	7	
	Oxford/AstraZeneca	3	6	3	2	
	Moderna	1	0			
Third dose						
	Pfizer/BioNtech	16	23	18	4	
	Moderna	1	0	1	4	
Fourth dose						
	Pfizer/BioNtech (Bivalent)		17			
	Pfizer/BioNtech		1			
	Moderna (Bivalent)		5			
Fifth dose						
	Pfizer/BioNtech (Bivalent)		1			
**Infection History**						
Infected		12	20	19	10	19
Naïve		5	3	0	0	0

B

	Variant infected	Date swab collected
**1**	BE.1_OMICRON	21/12/2022
**2**	CH.1.1.2_OMICRON	
**3**	CH.1.1_OMICRON	31/01/2023
**4**	BQ.1.1_OMICRON	04/01/2023
**5**		19/01/2023
**6**		21/12/2022
**7**		06/01/2023
**8**	BA.5.2-10	
**9**	BA.5.2-11	
**10**	BA.5.2-12	
**11**	BA.5.2-13	
**12**	BA.5.2-14	
**13**		24/01/2023
**14**		17/01/2023
**15**		17/01/2023
**16**	BE.1_OMICRON	14/08/2022
**17**	BA.2.73_OMICRON	16/09/2022
**18**	BA.5.1_OMICRON	09/10/2022
**19**	XBB.1.5_OMICRON	26/02/2023

Table S2. Additional Patient sample information. Related to STAR Methods.

Ab id.	Protein-Specific	Heavy chain				Light chain			
		Number of AA changes	V-GENE	J-GENE	D-GENE	Light Chain	Number of AA changes	V-GENE and allele	J-GENE and allele
XBB-1	RBD	14	1-69*01 F	2*01 F	3-22*01 F	k	11	4-1*01 F	1*01 F
XBB-2	RBD	11	3-53*01 F	4*02 F	5-12*01 F	k	7	1-33*01, or 1D-33*01 F	2*01 F
XBB-3	RBD	14	3-66*01 F	6*02 F	2-15*01 F	k	6	1-9*01 F	5*01 F
XBB-4	RBD	7	3-15*07 F	4*02 F	3-3*01 F	k	5	1-39*01,1D-39*01 F	4*01 F
XBB-5	RBD	11	3-72*01 F	6*01 F, or 6*02 F	4-17*01 F	k	13	1-39*01 F, or1D-39*01 F	4*01 F
XBB-6	RBD	8	3-9*01 F	6*02 F	6-19*01 F	λ	9	2-14*01 F	1*01 F
XBB-7	RBD	12	3-7*01 F	6*02 F	3-22*01 F	λ	8	2-14*03 F	2*01 F, or 3*01 F
XBB-8	RBD	9	3-53*01 F	6*02 F	1-1*01 F	k	6	1-33*01 F, or 1D-33*01 F	5*01 F
XBB-9	RBD	19	3-53*02 F	6*02 F or 4*03	1-26*01 F	k	13	1-33*01 F, or 1D-33*01 F	1*01 F, or 3*01 F
XBB-10	RBD	11	3-66*01 F	4*02 F	3-10*01 F	k	8	1-33*01 F, or 1D-33*01 F	2*04 F

Table S3. Ig variable gene usage for XBB mAbs. Related to Figures 4 and 6.

**Method**	X-ray Crystallography				Cryo-EM		
**Structure**	Delta-RBD/XBB-2/NbC1	Delta-RBD/XBB-6 /Beta-49	Delta-RBD/XBB-9 /Fab-Nb		BA.2.86-RBD/ ACE2 local refinement	BA.2.12.1-RBD/ XBB-4 local refinement	BA.2.86-RBD/ XBB-7 local refinement
**PDB/EMBD ID**	8QRG	8QRF	8R80		8QSQ, EMD-18639	8R8K, EMD-19002	8QTD, EMD-18649
**Data collection**							
Space group	$P2_12_12_1$	$P2_12_12$	$P2_12_12_1$	Voltage (kV)	300	300	300
Cell dimensions				Frames (EER fractions)	50	50	50
a, *b*, *c* (Å)	81.5, 104.6, 111.0	189.1, 146.5, 52.2	108.5, 126.4, 164.8	Dose rate (e$^-$/Å^2/s)	16.6	9.66	16.5
α, β, γ (°)	90, 90, 90	90, 90, 90	90, 90, 90	Total dose (e$^-$/Å^2)	50	50	50
Resolution (Å)	110–2.30 (2.34–2.30)[a]	63–3.70 (3.76–3.70)	66–4.03 (4.09–4.03)	Calibrated pixel size (Å^2)	0.7303	0.7303	0.7303
R_{merge}	0.088 (0.584)	0.780 (---)	0.386 (---)	Defocus (μm)	-2.6 to -0.8	-2.6 to -0.8	-2.6 to -0.8
R_{pim}	0.026 (0.327)	0.221 (2.812)	0.109 (1.300)	Movies	9633	6616	9069
$I/\sigma(I)$	17.9 (1.2)	4.8 (0.5)	5.4 (0.4)	Particles (final)	15,883	61,333	75,021
$CC_{1/2}$	0.999 (0.743)	0.948 (0.231)	0.995 (0.250)	Symmetry	C1	C1	C1
Completeness (%)	91.1 (52.7)	100 (99.2)	100 (99.1)	Map resolution (Å) Au-FSC = 0.143	3.7	3.4	3.6
Redundancy	10.5 (3.7)	13.3 (13.1)	13.4 (13.6)	Sharpening B-factor (Å^2)	-19.2	-69.0	-77.0
**Refinement**							
Resolution (Å)	49–2.30	58–3.70	66–4.03	Resolution (Å)	4.0	3.4	3.6
No. reflections	36754/1846	14920/828	18344/944	No. atoms	6513	4006	3220
R_{work} / R_{free}	0.182/0.228	0.272/0.317	0.268/0.313	*B* factors (Å^2)	451	69	61
No. atoms				r.m.s. deviations			
Protein	5750	8047	11366	Bond lengths (Å)	0.002	0.003	0.002
Ligand/ion/water	237	49		Bond angles (°)	0.4	0.5	0.5
B factors (Å^2)				Clash score	3.9	4.7	6.2
Protein	58	169	204	Ramachandran outlier (%)	0	0	0
Ligand/ion/water	51	140		Rotamer outlier (%)	1.15	0	2.6
r.m.s. deviations				d FSC model (0.5)	8.3	3.6	3.9
Bond lengths (Å)	0.005	0.003	0.002	CC (mask)	0.69	0.84	0.79
Bond angles (°)	0.7	0.6	0.5				

Table S4. Data collection, structure determination and refinement statistics. Related to Figures 5 and 6.
